# Large-scale external validation and comparison of prognostic models: an application to chronic obstructive pulmonary disease

**DOI:** 10.1186/s12916-018-1013-y

**Published:** 2018-03-02

**Authors:** Beniamino Guerra, Sarah R. Haile, Bernd Lamprecht, Ana S. Ramírez, Pablo Martinez-Camblor, Bernhard Kaiser, Inmaculada Alfageme, Pere Almagro, Ciro Casanova, Cristóbal Esteban-González, Juan J. Soler-Cataluña, Juan P. de-Torres, Marc Miravitlles, Bartolome R. Celli, Jose M. Marin, Gerben ter Riet, Patricia Sobradillo, Peter Lange, Judith Garcia-Aymerich, Josep M. Antó, Alice M. Turner, Meilan K. Han, Arnulf Langhammer, Linda Leivseth, Per Bakke, Ane Johannessen, Toru Oga, Borja Cosio, Julio Ancochea-Bermúdez, Andres Echazarreta, Nicolas Roche, Pierre-Régis Burgel, Don D. Sin, Joan B. Soriano, Milo A. Puhan, for the 3CIA collaboration

**Affiliations:** 1 Epidemiology, Biostatistics and Prevention Institute, University of Zurich, Zurich, Switzerland; 2 Department of Pulmonary Medicine, Kepler Universitatsklinikum GmbH, Linz, Austria; 3 Faculty of Medicine, Johannes Kepler Universitat Linz, Linz, Austria; 4 Facultad de Medicina UASLP, Universidad Autonoma de San Luis Potosi, San Luis Potosi, Mexico; 5 Dartmouth College Geisel School of Medicine, Dartmouth, NH USA; 6 Department of Pulmonary Medicine, Paracelsus Medizinische Privatuniversitat, Salzburg, Austria; 7 Hospital Universitario de Valme, Sevilla, Spain; 8 Internal Medicine, Hospital Universitario Mutua de Terrassa, Terrassa, Spain; 9 Pulmonary Department and Research Unit, Hospital Universitario NS La Candelaria, Tenerife, Spain; 10 Network and Health Services Research Chronic Diseases (REDISSEC), Hospital Galdakao, Bizkaia, Spain; 11 Servicio de Neumología, Hospital Universitari Arnau de Vilanova, Lleida, Spain; 12 Pulmonary Department, Clinica Universidad de Navarra, Pamplona, Spain; 13 European Respiratory Society (ERS) Guidelines Director, Barcelona, Spain; 14 Pulmonary and Critical Care Medicine, Brigham and Women’s Hospital, Boston, MA USA; 15 IISAragón and CIBERES, Hospital Universitario Miguel Servet, Zaragoza, Spain; 16 Department of General Practice, Academic Medical Center, University of Amsterdam, Amsterdam, The Netherlands; 17 Hospital Univarsitario de Cruces, Barakaldo, Vizcaya Spain; 18 Department of Public Health, Section of Social Medicine, University of Copenhagen, Copenhagen, Denmark; 19 ISGlobal, CIBER Epidemiología y Salud Pública (CIBERESP), Universitat Pompeu Fabra (UPF), Barcelona, Spain; 20 ISGlobal, Centre for Research in Environmental Epidemiology (CREAL), IMIM (Hospital del Mar Medical Research Institute, Universitat Pompeu Fabra (UPF), CIBER Epidemiología y Salud Pública (CIBERESP), Barcelona, Spain; 21 Institute of Applied Health Research, University of Birmingham, Birmingham, UK; 22 Division of Pulmonary and Critical Care, University of Michigan, Ann Arbor, MI USA; 23 Department of Public Health and Nursing, Norvegian University of Science and Technology, Trondheim, Norway; 24 Centre for Clinical Documentation and Evaluation, Northern Norway Regional Health Authority, Bodø, Norway; 25 University of Bergen, Haukeland University Hospital, Bergen, Norway; 26 Department of Global Public Health and Primary Care, University of Bergen, Bergen, Norway; 27 Department of Respiratory Care and Sleep Control Medicine, Graduate School of Medicine, Kyoto University, Kyoto, Japan; 28 Department of Respiratory Medicine, Hospital Son Espases-IdISBa-CIBERES, Palma de Mallorca, Spain; 29 Instituto de Investigación Sanitaria Princesa (IISP)-Servicio de Neumología- Hospital Universitario de la Princesa, Universidad Autónoma de Madrid, Madrid, Spain; 30 Universidad Nacional de la Plata, Hospital San Juan de Dios de La Plata, Buenos Aires, Argentina; 31 Hopitaux Universitaires Paris Centre, Service de Pneumologie AP-HP, Paris, France; 32 Hopital Cochin; Universite Paris Descartes, Paris, France; 33 University of British Columbia, James Hogg Research Centre, Vancouver, Canada; 34 Instituto de Investigación del Hospital Universitario de la Princesa (IISP), Universidad Autónoma de Madrid, Servicio de Neumología, Madrid, Spain; 35 Scientific and Methodological Consultant of SEPAR www.separ.es, Barcelona, Spain; 36 Epidemiology, Biostatistics and Prevention Institute, University of Zurich, Hirschengraben 84, Room HRS G29, CH -8001 Zurich, Switzerland; 37 Epidemiology & Department of Epidemiology, Johns Hopkins Bloomberg School of Public Health, Baltimore, MD USA

**Keywords:** COPD, Prognostic scores, Large-scale external validation, Performance comparison, Network meta-analysis

## Abstract

**Background:**

External validations and comparisons of prognostic models or scores are a prerequisite for their use in routine clinical care but are lacking in most medical fields including chronic obstructive pulmonary disease (COPD). Our aim was to externally validate and concurrently compare prognostic scores for 3-year all-cause mortality in mostly multimorbid patients with COPD.

**Methods:**

We relied on 24 cohort studies of the COPD Cohorts Collaborative International Assessment consortium, corresponding to primary, secondary, and tertiary care in Europe, the Americas, and Japan. These studies include globally 15,762 patients with COPD (1871 deaths and 42,203 person years of follow-up). We used network meta-analysis adapted to multiple score comparison (MSC), following a frequentist two-stage approach; thus, we were able to compare all scores in a single analytical framework accounting for correlations among scores within cohorts. We assessed transitivity, heterogeneity, and inconsistency and provided a performance ranking of the prognostic scores.

**Results:**

Depending on data availability, between two and nine prognostic scores could be calculated for each cohort. The BODE score (body mass index, airflow obstruction, dyspnea, and exercise capacity) had a median area under the curve (AUC) of 0.679 [1st quartile–3rd quartile = 0.655–0.733] across cohorts. The ADO score (age, dyspnea, and airflow obstruction) showed the best performance for predicting mortality (difference AUC_ADO_ – AUC_BODE_ = 0.015 [95% confidence interval (CI) = −0.002 to 0.032]; *p* = 0.08) followed by the updated BODE (AUC_BODE updated_ – AUC_BODE_ = 0.008 [95% CI = −0.005 to +0.022]; *p* = 0.23). The assumption of transitivity was not violated. Heterogeneity across direct comparisons was small, and we did not identify any local or global inconsistency.

**Conclusions:**

Our analyses showed best discriminatory performance for the ADO and updated BODE scores in patients with COPD. A limitation to be addressed in future studies is the extension of MSC network meta-analysis to measures of calibration. MSC network meta-analysis can be applied to prognostic scores in any medical field to identify the best scores, possibly paving the way for stratified medicine, public health, and research.

**Electronic supplementary material:**

The online version of this article (10.1186/s12916-018-1013-y) contains supplementary material, which is available to authorized users.

## Background

Prognostic scores, commonly based on coefficients from regression models, provide a probability of a certain adverse outcome for an individual over a specified time horizon. Prognostic scores have become increasingly popular over the last two decades [1–5]. They serve multiple purposes such as informing individuals and health care providers about disease and outcome risks, supporting risk-stratified and personalized prevention or treatment decisions, identifying participants for research, or adjusting for confounding [6–9].

Numerous prognostic models have been developed in various fields of medicine [10–13]. Just for predicting the risk of cardiovascular disease in the general population, a recent review identified 363 prognostic models or scores [14]. For patients with chronic obstructive pulmonary disease (COPD), prognostic scores have been developed mostly to predict the risk of death [15–30], but scores also exist to predict exacerbations [31] or deteriorating of health-related quality of life [27, 32].

Major obstacles for using prognostic scores in practice and research are, however, the frequent lack of external validations, comparisons of their predictive performance, and assessments of their applicability in practice [2, 33–38]. Practitioners and researchers are left with uncertainty about which prognostic score to use and may be reluctant to use them at all [39]. Ideally, prognostic scores would be externally validated in several different populations and their performance summarized [40, 41]. However, such external validations and concurrent comparisons are rarely performed [42]. In addition, for even more comprehensive comparison, the performance of prognostic scores may be compared indirectly using common comparator scores similar to network meta-analysis (NMA) [43–48] of randomized trials.

Our aim was to use multiple score comparison (MSC) in order to externally validate and concurrently compare prognostic scores for 3-year mortality in patients with COPD.

## Methods

We followed a prespecified study protocol and described the detailed statistical methods elsewhere [43].

### Study design and participants

This study was based on 26 cohort studies of the COPD Cohorts Collaborative International Assessment (3CIA) consortium. Details have been reported elsewhere (and summarized in Table 2) [49]. All cohorts were approved by ethics committees, and participants gave written informed consent [49]. We also included the Phenotype and Course (PAC)-COPD and Copenhagen cohorts in the final database, even if they were used in the large-scale update of the ADO (age, dyspnea, and airflow obstruction) index [15]. We considered this approach reasonable, since they form only a small part of the final database, but we verified in a sensitivity analysis if they affected the results.

### Prognostic scores

Starting from the literature review of two studies [32, 42] and searching among their references, PubMed-related articles, and through our research network, we identified 19 prognostic scores, of which we included 10 in our analysis. The scores (see Table 1 for details) were the BODE (body mass index, airflow obstruction, dyspnea, and severe exacerbations) [17], updated BODE [16], ADO ( we included in the analysis only the updated ADO index and not the original ADO index [16] because the updated ADO was generated from large-scale external validation; however, we will name it simply ADO) [15], eBODE (severe acute exacerbation of COPD plus BODE) [18], BODEx (body mass index, airflow obstruction, dyspnea, severe acute exacerbation of COPD) [18], DOSE (dyspnea, obstruction, smoking and exacerbation frequency) [27], SAFE (Saint George’s Respiratory Questionnaire (SGRQ) score, air-flow limitation and exercise capacity) [28], and B-AE-D (body mass index, acute exacerbations, dyspnea; we used the optimized version and not the original B-AE-D score) [23]. The Global Initiative for Chronic Obstructive Lung Disease (GOLD) classification [50, 51] and the 2011–2016 GOLD classification (often referred to as new GOLD in the recent COPD literature) [51] were also used in the analysis, even if they were not designed for prognostic purposes. Apart from original ADO and original B-AE-D score the other seven identified scores from the literature were excluded from the analysis, since our database did not include at least one of their predictors or did not include them simultaneously in at least one cohort.

**Table 1:** Scoring rules of prognostic scores to predict mortality in patients with COPD

Score Predictor	GOLD [50, 51]	GOLD (2011–2016) [51]	BODE [17]	BODE upd. [16]	ADO [15]	e-BODE [18]	BODEx [18]	DOSE [27]	SAFE [28]	B-AE-D [23]
BMI			0 (> 21)	0 (> 21)		0 (> 21)	0 (> 21)			0 (> = 21)
			1 (<= 21)	1 (<= 21)		1 (<= 21)	1 (<= 21)			6 (18.5–21)
										9 (< 18.5)
FEV1% pred.	0 (> = 80%)	0 (if FEV1pp > = 50 and <= 1 exacerbations per year)	0 (> = 65%)	0 (> = 65%)	0 (> = 81%)	0 (> = 65%)	0 (> = 65%)	0 (> = 50%)	0 (> = 80%)	
	1 (50–79%)	2 (otherwise)	1 (50–64%)	1 (36–64%)	1 (65–60%)	1 (50–64%)	1 (50–64%)	1 (31–49%)	1 (50–79%)	
	2 (30–49%)		2 (36–49%)	2 (<= 35)	2 (51–64%)	2 (36–49%)	2 (36–49%)	2 (<= 30)	2 (36–49%)	
	3 (< 30%)		3 (<= 35)		3 (35–50%)	3 (<= 35)	3 (<= 35)		3 (<= 35)	
					4 (<= 35%)					
mMRC		0 (if mMRC > = 2 and CAT > = 10)	0 (0–1)	0 (0–1)	0 (0)	0 (0–1)	0 (0–1)	0 (0–1)		0 (0–2)
		1 otherwise	1 (2)	1 (2)	1 (1–2)	1 (2)	1 (2)	1 (2)		6 (3)
			2 (3)	2 (3)	2 (3)	2 (3)	2 (3)	2 (3)		10 (4)
			3 (4)	3 (4)	3 (4)	3 (4)	3 (4)	3 (4)		
6MWT (m)			0 (> = 350)	0 (> = 350)		0 (> = 350)			0 (> = 400)	
			1 (250–349)	4 (250–349)		1 (250–349)			1 (300–399)	
			2 (150–249)	7 (150–249)		2 (150–249)			2 (200–299)	
			3 (<= 149)	9 (<= 149)		3 (<= 149)			3 (<= 199)	
Age (years)					0 (40–49)					
					2 (50–59)					
					4 (60–69)					
					5 (70–79)					
					7 (> = 80)					
Prev. exacerbation		(See FEV1pp)				0 (0)	0 (0)	0 (0–1)		0 (0)
						1 (1–2)	1 (1–2)	1 (2–3)		3 (1)
						2 (> 2)	2 (> 2)	2 (> 3)		7 (> = 2)
CAT		(See mMRC)								
Smoking								0 (non- smoker)		
								1 (current smoker)		
Quality of life (SGRQ)									0 (<= 30)	
									1 (31–49)	
									2 (50–64)	
									3 (> = 65)	
Total score	0–3	0–3	0–10	0–15	0–14	0–12	0–9	0–8	0–9	0–26

*Abbreviations: BMI* body mass index, *FEV1% pred.* forced expiratory volume in 1 s percentage predicted, *mMRC* modified Medical Research Council dyspnea scale, *6MWT* 6-min walk test, *CAT COPD Assessment Test*, *SGRQ* Saint George’s Respiratory Questionnaire; previous exacerbations are referred to the previous year, *GOLD* Global Initiative for Chronic Obstructive Lung Disease, *BODE* body mass index, airflow obstruction, dyspnea and severe exacerbations, *BODE upd.* BODE updated, *ADO* age, dyspnea, airflow obstruction (we use in our analysis the updated version of the ADO score), *e-BODE* severe acute exacerbation of COPD plus BODE, *BODEx* body mass index, airflow obstruction, dyspnea, severe acute exacerbation of COPD, *DOSE* dyspnea, obstruction, smoking, and exacerbation frequency, *SAFE* Saint George’s Respiratory Questionnaire (SGRQ) score, air-flow limitation and exercise capacity, *B-AE-D* body mass index, acute exacerbations, dyspnea (we use the optimized version of the score, introduced in the same paper). Missing cells correspond to variables that do not constitute the score of the correspondent column

### Outcome and performance measure for external validation and comparison of prognostic scores

We evaluated a number of performance measures commonly used to assess the prognostic properties of prediction models and scores [43]. We deemed the area under the curve (AUC) to be the most appropriate performance measure for our purposes, mainly because its range is independent of the data, it is easy to interpret, and an analytic formula for its variance is available [52].

### Statistical analysis

We followed a prespecified study protocol. We first performed direct head-to-head comparisons using random effects meta-analysis and then examined the network evidence merging all available direct and indirect evidence [53]. We used a novel methodology, i.e., MSC meta-analysis, adapted from multiple treatment comparison network meta-analysis [54, 55]. Methodological details are reported in the section “Detailed Methods” in Additional file 1 and in a recent paper [43]. R codes are available (provided in the section “R Code for MSC meta-analysis” in Additional file 1).

### Direct comparisons (random effects pairwise meta-analysis)

We directly compared prognostic scores by pairwise random effects meta-analysis [56, 57]. We used forest plots to visually investigate statistical heterogeneity as well as the *I*^*2*^ statistic. Such standard meta-analysis has limitations, since it does not take into account the correlations among multiple scores evaluated on the same set of patients [58], and it does not give a clear indication of which prognostic score performs best. Thus, we adopted network meta-analysis, an approach that allowed us to weight and then pool the results coming from different cohorts.

### MSC meta-analysis

Methodological details are reported in detail in [43]. In brief, we used an example of implementation of network meta-analysis for treatment effectiveness comparison [54], adapting it to our purposes, namely to concurrently externally validate and compare prognostic scores from individual patient data across different cohorts [43]. We have explicitly included correlations [58] between the scores on a cohort level. We use a frequentist two-stage meta-regression model, as proposed in [54]:

1. Ordinary meta-analysis (stage I) to obtain the direct estimates for pooled differences in AUC (using the inverse-variance weighted means of the corresponding cohorts). The meta-analyses were done within each group of cohorts where data for the same prognostic scores were available.
2. In stage II, we merged the estimates for the differences in AUC from the groups of cohorts, looking for the weighted least squares solution to the regression problem equation. Based on the direct estimates and their variances from the first stage, we estimated the pooled differences in AUC that obeyed fundamental consistency equations. Thus in stage II, the stage I estimates for the differences in AUC were combined across groups of cohorts to give overall performance estimates for the entire network.

In order to provide a ranking of the scores, we used a frequentist version of the surface under the cumulative ranking curve (SUCRA) [59, 60] score showing the likelihood of the score to be better than any other score and summarizing relative performances and confidence intervals.

The last steps were to ensure that the heterogeneity, transitivity, and consistency assumptions were met [46]. Heterogeneity in the MSC analysis was evaluated by the pooled heterogeneity variance among groups (*τ*^*2*^_*pooled*_*)*. We assessed “transitivity” through analysis of variance (ANOVA) tests. Thus, we assessed the comparability of the cohorts across whom the predictive performance of a score may vary because of a “spectrum effect” [61] or “case mix” [37, 62, 63]. We also assessed consistency [46] between direct evidence and MSC meta-analysis estimates using the Q likelihood-ratio test statistic to evaluate the global consistency and analysis of residuals and leverages to evaluate the local consistency [54]. For more details, see “Detailed Methods” in Additional file 1 and [43].

### Handling of missing data

If a variable was missing for > 30% of the patients, we discarded the specific variable for that particular specific cohort, since the effects of such predictors could be generally distrusted [1]. Otherwise we performed multiple imputation with chained equations (the analysis of the patterns of missingness allowed us to consider the missing data missing completely at random apart from the dependence on the cohort) [4]. We combined the estimates of the 30 different analyses (one for each imputed dataset, for each of which we followed all the previously highlighted frequentist two-stage meta-regression model approaches) using Rubin’s rules.

## Results

### Cohort and participant characteristics

The cohorts varied greatly in terms of geographic location, sample size, and number of events and included a broad spectrum of patients with COPD from primary, secondary, and tertiary care settings (Table 2). Mean forced expiratory volume in 1 s percentage (FEV1) ranged from 30 to 70% of the predicted values, mean modified Medical Research Council (mMRC) dyspnea scores from 1.0 to 2.8 (the scale goes from 0 to 4, with 4 being the worst), mean number of exacerbations in the previous year (where available) from 0.2 to 1.7, and mean 6-min walk distance (where available) from 218 to 487 m.

**Table 2:** Study characteristics

Cohort	Number of Events	Number of patients	Person years	Mean age, years	Men, %	Mean FEV1%pred.	Mean mMRC	Past exacerbators, %	Mean no. prev. exacerbations	Current smoker, %	Mean BMI	Mean 6MWT, m	Mean SGRQ	Mean CAT
COPDgene	337	4484	10,603	63 (9)	56	57.4 (22.8)	1.5	0.16		43	27.9 (6.1)	376.1 (124.1)	36.9 (22.9)	
Sevilla^a^	205	596	1562	66 (10)	95	43.5 (13.3)	1	0.25	1.16	24	29.2 (5.7)			
Copenhagen^b^	186	2287	6618	61 (9)	54	70.5 (23.7)	1.3			71	25 (4.2)			
Genkols	126	954	2708	65 (10)	61	46.9 (17)	1.3	0.15	0.6	47	25.4 (5)			
Zaragoza II^a^	118	1150	3069	63 (9)	93	62.3 (20.3)	1.1	0.17	0.91	34	27.5 (4.8)	356.2 (153.7)		
HUNT	116	1571	4583	63 (13)	62	63.8 (18.7)	1.3			47	26.4 (4.4)			
Galdakao^a^	92	543	1497	68 (8)	96	55 (13.3)	0.9	0	0.65	21	28.3 (4.4)	408.9 (92.4)		
Barmelweid^b^	79	232	555	72 (9)	60	45.2 (16.1)	1.1			21	26 (6.3)	363.4 (126.8)		
Terrassa III^a^	78	181	423	72 (10)	95	45.2 (14.4)	1.2	0.31	1.28	23	27.9 (5)	330.4 (105.8)		
Initiatives BPCO	76	930	1525	64 (10)	77	52.4 (20.3)	1.1	0.4	1.65	28	25.4 (5.5)	387.4 (120.8)	43.9 (19)	
Terrassa I^a^	72	135	284	72 (9)	92	41.3 (13)	1.3	0.25	1.03	17	26.3 (4.9)			
SEPOC^b^	61	318	871	65 (9)	100	45 (18.3)	1.5			38	26.4 (4.2)			
Requena II^a,c^	52	186	396	71 (9)	99	44.5 (16.5)	1	0.16	0.62	17	28.1 (5.2)	380.1 (111.9)		
ICE COLD ERIC	47	400	1071	67 (10)	57	55.3 (16.5)	1.5	0.13	0.58	39	26.1 (5.2)			
PAC-COPD^b^	41	342	980	68 (9)	93	52.4 (16.2)	1	0.04		33	28.2 (4.7)	435.5 (90.6)		
Tenerife^a^	34	275	653	63 (10)	79	55.8 (21.2)	1.2	0.06	0.37	42	27.3 (5.1)	487.4 (87.5)		
Terrassa II^a^	28	66	145	72 (9)	98	30.2 (12.9)	1	0.42	1.81	14	25.7 (4.3)	217.7 (76.6)		
Requena I^a^	23	174	393	72 (9)	99	48.1 (16.8)	1.2^c^	0.03	0.22	23	28 (4.2)	434.4 (125.3)		
Zaragoza I^a^	21	137	379	66 (8)	99	49.8 (17.6)	1.1			27	27.7 (4.6)	449 (91.9)		
Son Espases Mallorca	17	115	292	70 (7)	79	41.5 (13.4)	1	0.59		27	27.1 (5.9)	401.5 (89.7)		16.6 (8.2)
Basque^b^	16	106	299	71 (9)	98	46.9 (11.4)	0.6			23	26.1 (4.9)	442.9 (95.4)		
Japan	15	147	409	69 (7)	100	47.1 (17.5)	0.9			22	21 (2.9)		36.6 (16.5)	
La Princesa Madrid	11	318	633	71 (10)	74	50 (19.8)	1.1	0.18	0.77	19	26.2 (5.1)	337.1 (92.8)		
Pamplona^a^	7	190	470	65 (8)	84	68.9 (19.9)	1.1			37	27 (4.4)	463.2 (113.9)		
Mar de Plata Argentina	3	99	147	64 (9)	60	48.8 (18.6)	1	0.29		21	27 (5.6)	353.2 (128.7)		16.1 (7.8)
A1ATD^d^	0	308	834	58 (10)	60	53.1 (25.1)	1.2	0.52		5	25.7 (4.9)		50.8 (19.9)	20.5 (8.1)

*Abbreviations: FEV1% pred.* forced expiratory volume in 1 s percentage predicted, *mMRC* modified Medical Research Council (*MMRC*) dyspnea scale; past exacerbators are defined as patients with more than one exacerbation in the previous year; mean number previous exacerbations are referred to the previous year, *BMI* body mass index, *6MWT* 6-min walk test, *SGRQ* Saint George’s Respiratory Questionnaire, *CAT COPD Assessment Test*

The cohorts are presented in decreasing order of number of events. Most of the variables available provided by the 3CIA collaboration for the different cohorts are shown. In particular, we show all the variables constituting the scores analyzed in our study. We present the standard deviation for all individual variables whose distribution is approximately normal; this is not the case for count (with small numbers) or categorical variables, like number of previous exacerbations or mMRC)

^a^Cohorts belonging to the Collaborative Cohorts to Assess Multicomponent Indices of COPD in Spain (COCOMICS) collaboration

^b^Cohorts belonging to the ADO collaboration. For information concerning the cohorts, see [49]

^c^Since none of the scores could be evaluated in the cohort Requena I (mainly because the variable dyspnoea was missing for 95% of the patients, i.e., for 165 out of 174 patients), this cohort was excluded from the analysis

^d^Since there was no event in a follow-up of 3 years, the cohort A1ATD was excluded from the analysis

Missing cells correspond to variables that are completely missing in the cohort of the correspondent row

### Direct comparisons of prognostic scores for mortality in patients with COPD

The direct comparisons are shown in the upper-right triangle of Table 3, i.e., a league table (that also includes the MSC meta-analysis in the lower-left triangle). Forty-one direct comparisons of the AUC of prognostic scores were possible; indeed, no direct evidence was available for the comparison between SAFE and the eBODE, BODEx, DOSE, and B-AE-D scores (cells D6, E6, F2, G6, I10 in the league Table 3).

**Table 3:** League table presenting the multiple score comparison (MSC) meta-analysis (lower-left half of the table) and the direct random effects meta-analysis (upper-right half of the table)

Direct meta- analysis MSC meta- analysis		GOLD	B-AE-D	GOLD (2011–2016)	DOSE	BODEx	SAFE	eBODE	BODE	BODE updated	ADO
		1	2	3	4	5	6	7	8	9	10
GOLD	A	**AUC = 0.613 (1st Qu. 0.587, 3rd Qu. 0.637)**	ΔAUC = 0.030 (−0.005, 0.065)	0.017 (−0.005, 0.038)	0.036 (0.008, 0.064)	0.054 (0.029, 0.079)	0.047 (0.027, 0.068)	0.064 (0.004, 0.123)	0.071 (0.040, 0.102)	0.080 (0.041, 0.119)	0.090 (0.072, 0.109)
B-AE-D	B	ΔAUC = 0.010 (−0.010, 0.031)		−0.004 (−0.025, 0.017)	0.004 (− 0.012, + 0.020)	0.025 (0.011, 0.039)	NA	0.069 (−0.016, −0.121)	0.079 (0.004, 0.154)	0.082 (−0.012, −0.152)	0.076 (0.051, 0.101)
GOLD (2011–2016)	C	0.012 (−0.001, 0.024)	0.001 (− 0.019, 0.021)		0.009 (− 0.002, 0.021)	0.028 (0.017, 0.039)	0.055 (0.038, 0.072)	0.047 (0.016, 0.079)	0.059 (0.046, 0.073)	0.051 (0.027, 0.076)	0.067 (0.053, 0.080)
DOSE	D	0.022 (0.006, 0.037)	0.011 (−0.007, − 0.029)	0.010 (− 0.005, 0.025)		0.018 (0.008, −0.029)	NA	0.039 (0.007, 0.070)	0.033 (−0.000, 0.065)	0.043 (−0.002, 0.088)	0.061 (−0.044, −0.079)
BODEx	E	0.041 (0.027, 0.055)	0.030 (0.014, 0.047)	0.029 (0.015, 0.043)	0.019 (0.005, 0.033)		NA	0.030 (−0.001, 0.061)	0.031 (−0.017, 0.079)	0.039 (−0.028, 0.105)	0.050 (0.034, 0.066)
SAFE	F	0.061 (0.034, 0.087)	0.050 (0.018, 0.082)	0.049 (0.022, 0.076)	0.039 (0.010, 0.068)	0.020 (−0.009, 0.048)		NA	0.011 (−0.000, 0.023)	0.005 (−0.009, 0.018)	−0.007 (−0.029, 0.015)
eBODE	G	0.065 (0.046, 0.085)	0.055 (0.032, 0.078)	0.054 (0.034, 0.074)	0.044 (0.023, 0.064)	0.024 (0.007, 0.042)	0.005 (−0.025, 0.035)		−0.001 (−0.020, 0.017)	0.002 (−0.031, 0.034)	0.024 (−0.018, 0.066)
BODE	H	0.068 (0.052, 0.084)	0.057 (0.034, 0.080)	0.056 (0.039, 0.074)	0.046 (−0.027, −0.065)	0.027 (0.009, 0.045)	0.007 (−0.019, 0.034)	0.003 (−0.016, 0.021)		0.005 (−0.006, 0.017)	−0.004 (−0.023, 0.016)
BODE upd.	I	0.076 (0.058, 0.095)	0.066 (0.041, 0.091)	0.065 (0.045, 0.085)	0.055 (−0.033, −0.076)	0.036 (0.015, 0.056)	0.016 (−0.012, 0.043)	0.011 (−0.009, 0.031)	0.008 (−0.005, 0.022)		−0.005 (−0.032, 0.022)
ADO	L	0.083 (0.070, 0.096)	0.072 (0.052, 0.093)	0.071 (0.056, 0.087)	0.070 (−0.052, −0.089)	0.042 (0.026, 0.058)	0.022 (−0.005, 0.050)	0.018 (−0.003, 0.038)	0.015 (−0.002, 0.032)	0.007 (−0.012, 0.026)	

*Abbreviations: AUC* area under the curve; the lower-left half of the table refers to the MSC meta-analysis. The upper-right half of the table refers to direct comparisons using conventional random effects meta-analysis. The first cell (first row, first column) gives a reference value (in boldface), namely the median and 1st and 3rd quartiles of the AUC of the GOLD classification across cohorts as an anchor to interpret the differences in AUC between the prognostic scores. In every other cell, each pair of scores is compared using the difference in AUC. Lower-left half of the table we report in the correspondent cell the difference between the AUCs of the score in the row and the score in the column; instead, for the upper-right half of the table we report the difference between the AUCs of the score in the column and the score in the row or the. We decided for this representation to make a visual comparison between direct and MSC comparison easier; in this way, it is enough to look at corresponding values mirrored at the main diagonal. The 95% confidence interval is indicated in parentheses. For better readability of the table the sign “+” is omitted, while the sign “–” is indicated

The updated BODE score performed statistically significantly better than GOLD, new GOLD, and the B-AE-D scores, whereas the AUC of the updated BODE score was higher than for the other scores but not statistically significantly so. We deemed overall statistical heterogeneity of direct comparisons moderate. However, in our MSC meta-analysis the direct comparisons should be interpreted with caution, since they do not take into account that multiple scores were evaluated on the same set of patients and are thus likely to bias the interpretation of which prognostic score performs best [58].

### Groups of cohorts evaluating the same prognostic scores

**Fig. 1:**
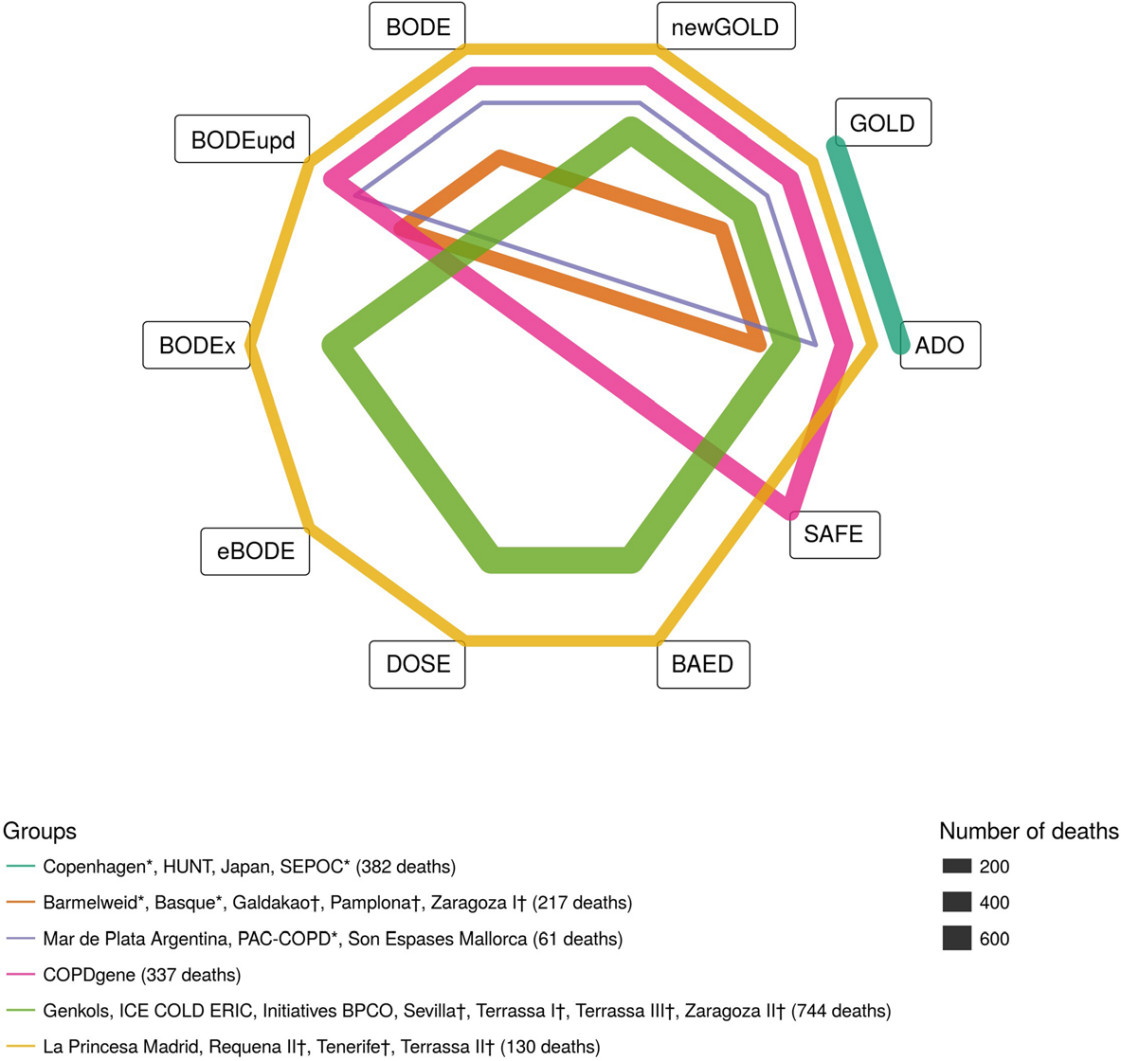
Network plot. Network representing which prognostic scores belong to the different groups. Each node represents a score and each closed polygon represents a group of cohorts where the same prognostic scores are available. The thickness of the lines represents the total number of deaths in the specific group

Grouping of cohorts where the same prognostic scores could be calculated was the first step to consider correlations introduced by predictions performed on the same sample of patients. Figure 1 shows the grouping of cohorts. In group 1 (constituting four cohorts: Copenhagen, HUNT, Japan, SEPOC, as shown in Fig. 1) information on FEV1, age, and dyspnea was available to calculate the GOLD and ADO scores for each participant. In contrast, group 6 consisted of four cohorts (La Princesa Madrid, Requena II, Tenerife, Terrassa II) where nine prognostic scores (all except for the SAFE score) could be calculated for each participant. Figure 1 provides a visual representation of these groups together with the number of events (i.e., deaths). For example, the dark green line represents group 1 where the GOLD and ADO scores could be compared against each other. The closed polygons show the comparisons that are possible for each group of cohorts. Group 6 is represented by the dark yellow polygon that includes nine scores. Thus, unlike multiple treatment network meta-analyses, where usually two or at most three treatments are compared in each trial, Fig. 1 shows that in each of the cohorts of our database we can compare between two and nine prognostic scores.

### MSC meta-analysis of prognostic scores to predict 3-year mortality in patients with COPD

The lower-left part of Table 3 shows all comparisons between the AUCs of the 10 prognostic scores taking into account the correlation among multiple comparisons for the same patients as well as direct and indirect evidence of the entire network (Fig. 1). The median AUC of the GOLD classification of airflow obstruction severity was 0.613 (interquartile range 0.587 to 0.637) and is shown in boldface in the upper-left cell as an anchor to interpret the differences in AUC between the prognostic scores. Compared to GOLD, all prognostic scores showed statistically significantly higher AUCs except for the B-AE-D and GOLD 2011–2016 (cells B1-L1 in Table 3). Compared to the BODE score (the most commonly used prognostic score in COPD, median AUC 0.679 [interquartile range 0.655 to 0.733]), the ADO, updated BODE, and eBODE showed higher AUCs, whereas all other scores performed worse.

**Fig. 2:**
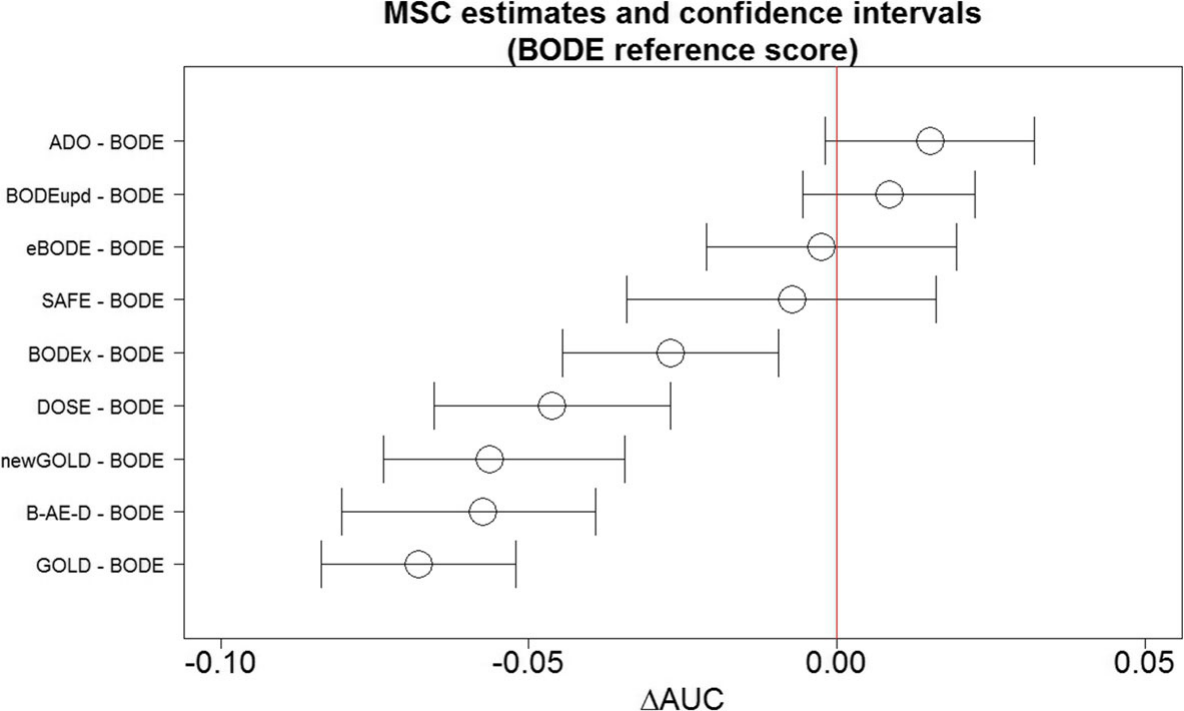
Comparison of AUC of prognostic scores. Difference in AUC (shown with confidence interval with 95% confidence level) among the different scores and the BODE index (chosen here as the reference score) in the MSC meta-analysis. As a reference we use the median of the AUC of the BODE score 0.679 (1st Qu. 0.655, 3rd Qu. 0.733)

Figure 2 shows the comparisons of all scores against the BODE score and that the ADO score and the updated BODE performed better than the other scores (i.e., AUC_ADO_ – AUC_BODE_ = +0.015 [95% CI –0.002 to 0.032], *p* = 0.08; AUC_BODE updated_ – AUC_BODE_ = 0.008 [95% CI = −0.005 to +0.022]; *p* = 0.23). The sensitivity analysis undertaken excluding from the database the two cohorts used in the large-scale update of the ADO index [15] shows no significant differences.

### Heterogeneity, transitivity, and inconsistency

Global heterogeneity was relatively small (*τ*^*2*^_*pooled*_ = 0.00011) (we did not use a *τ*^*2*^ for each group (*τ*^*2*^_*g*_) since this is not recommended when there are groups with a single cohort [54]). The groups of the MSC meta-analysis were balanced with regard to characteristics of the different cohorts that may modify the predictive performance of the scores (all a priori defined characteristics that were generating case mix were not statistically significantly different across groups), and we could thus assume transitivity.

The consistency analyses did not suggest local or global inconsistency. Visual analysis of the Q-Q plot and studentized residuals indicated robust local consistency. The likelihood-ratio test statistic showed overall consistency (Q likelihood-ratio test = 25.29 ≅ *χ*^*2*^(0.95, 16) = 26.30, *p* value = 0.06).

## Discussion

Our study has two main findings. Firstly, our results indicate that the ADO index has the best ability to predict 3-year mortality in patients with COPD, followed by the updated BODE and eBODE indices. Given its simplicity, the ADO index may be the most attractive option across care settings to inform patients and health care professionals about prognosis and to inform treatment decisions whose effectiveness may depend on life expectancy. Secondly, we presented a comprehensive approach for external validation and concurrent comparison of prognostic scores and its first application. MSC meta-analysis is a method adapted from network meta-analysis that meets the call for new approaches for external validation and concurrent comparison of risk prediction models and scores that should take advantage of data sharing, individual patient data (IPD), and advanced analytical techniques [36, 37, 45, 64, 65].

In practice, the GOLD score using just lung function is still used most commonly to grade disease severity, which is traditionally related to prognosis as in other fields (e.g., cancer). FEV1% pred. (thus, GOLD classification) is an important parameter at the population level in the prediction of important clinical outcomes such as mortality and hospitalization. The revised combined COPD assessment and their further developments integrate the severity of airflow limitation assessment, also providing information regarding symptom burden and risk of exacerbation [51]. However, the results of our analysis show that, when the aim is to predict mortality in individuals, other scores such as ADO, updated BODE, and eBODE are substantially better than the GOLD classifications (in our analysis, GOLD and GOLD 2011–2016). We note that the AUC for the best score (ADO) is 0.69, a moderately good discriminative performance; however, we can often not expect a much higher discriminative performance in clinical settings (for instance, see [31]).

The predictive performance of a prognostic score is important, but it is not the only criterion for choosing a prognostic score for practice. Indeed, with an eye towards applicability, the time, cost, and burden for patients and practitioners to measure the predictors of a prognostic score should be taken into consideration [66]. We deem a prognostic score such as ADO to be easily available if it only includes simple questions, easily available information from medical charts, and spirometry (performed for the diagnosis of COPD) [50, 51].

Scores to predict mortality are also useful beyond estimating prognosis. Nowadays, no treatments to lower the risk of mortality are currently available for patients with COPD; thus, for this outcome, prediction scores cannot provide risk-stratified treatment guidance. However, prognostic scores may help to make randomized trials with all-cause mortality as primary outcome more efficient than previous trials by only including patients at higher risk [67]. Also, prognostic scores for all-cause mortality are particularly attractive for multimorbid patients such as COPD patients, where cardiovascular disease, diabetes, renal disease, and lung cancer*, among other conditions,* also contribute to mortality [68, 69]. Patients with COPD often receive less than optimal prevention and treatment of cardiovascular disease, which may partly reflect a therapeutic nihilism. Of course, there are patients who are unlikely to benefit from long-term cardiovascular prevention because of short life expectancy. However, a prognostic score provides a better basis for decisions on cardiovascular prevention, lung cancer screening, or other treatments and may limit under- and over-treatment in COPD [1, 70, 71].

Many prognostic models and scores (as in the models’ simplified forms) are never validated in practice, and many investigators develop a second model instead of relying on existing scores at least as a starting point. Such practice has led to numerous prognostic scores for the same conditions that are left without external validation. Thus, we introduced MSC meta-analysis, which addresses the lack of external validation and comparisons of prognostic scores by comparing their predictive performance in external validation cohorts and simultaneously considering the entire network of direct and indirect comparisons. Thereby, it allows for a comparison of predictive performance that is not limited by non-comparable spectrum of populations, as is commonly the case when evaluating the results of independent validation studies. MSC meta-analysis can be applied to any medical field, with the availability of individual patient data being the only major limiting factor.

Strengths of our study include the careful analytical approach to MSC meta-analysis and the availability of the R code, which allows for widespread use and potential further development of the method. For the particular application of MSC meta-analysis here, a major strength is the large high-quality database of the 3CIA collaboration with the broadest possible COPD patient spectrum. The diverse case mix and broad patient spectrum greatly increase the probability that our results are generalizable to all COPD patients. A limitation of the study is that, ideally, a network meta-analysis is conducted prospectively and jointly planned for all of the cohorts involved to ensure equality of the clinical settings and homogeneity of study design, conduct, and variable definitions, though this will rarely be the case in reality. Another limitation of our analysis is that we only used AUC as a performance measure, which we did for theoretical and practical reasons [43]. In general, improvements in AUC have to be interpreted with caution [72]. Furthermore, we cannot exclude the possibility of case-mix effects due to variables that were not available in the database or unknown.

Further research needs include the extension of MSC to include measures of calibration, which is arguably as important as discrimination. For the area of COPD, it would be attractive to apply MSC to risk scores for exacerbations [51, 73]. However, there are likely too few thoroughly developed and externally validated scores to predict exacerbations in patients with COPD [31]. Finally, given the large number of risk scores in the medical field and the lack of external validations and comparisons of risk scores, there is a great need for comparative studies that may use MSC in order to inform clinical practice and research about the most predictive scores [31].

## Conclusions

Borrowing from network meta-analysis, we presented a comprehensive approach for external validation and concurrent comparison of multiple prognostic scores. While our analyses showed best performance for the ADO and updated BODE scores to predict mortality for patients with COPD, MSC meta-analysis can be applied to prognostic scores in any medical field to identify the best scores, possibly paving the way for stratified medicine, public health, and research.

## Additional files

Additional file 1: The Appendix. (DOCX 90 kb)

## Abbreviations

ADO: Age, dyspnea, airflow obstruction
AUC: Area under the curve
B-AE-D: Body mass index, acute exacerbations, dyspnea
BODE: Body mass index, airflow obstruction, dyspnea and severe exacerbations
BODEx: Body mass index, airflow obstruction, dyspnea, severe acute exacerbation of COPD
COPD: Chronic obstructive pulmonary disease
DOSE: Dyspnea, obstruction, smoking and exacerbation frequency
e-BODE: Severe acute exacerbation of COPD plus BODE
GOLD: Global Initiative for Chronic Obstructive Lung Disease
MSC: Multiple score comparison
NMA: Network meta-analysis
SAFE: Saint George’s Respiratory Questionnaire (SGRQ) score, air-flow limitation and exercise capacity
SUCRA: Surface under the cumulative ranking curve
