# Supplementary material for: Large-scale external validation and comparison of prognostic models: an application to chronic obstructive pulmonary disease
Source: BMC Med. 2018 Mar 2;16:33. doi: 10.1186/s12916-018-1013-y (PMC5833113; doi:10.1186/s12916-018-1013-y)
Supplement: Supplementary file 1 — The Appendix. (DOCX 90 kb) [file 12916_2018_1013_MOESM1_ESM.docx]

**A novel comprehensive approach for large-scale external validation and comparison of prognostic models: An application to chronic obstructive pulmonary disease**

Guerra et al. BMC Medicine

https://doi.org/10.1186/s12916-018-1013-yhttps://doi.org/10.1186/s12916-018-1013-y

TABLE of CONTENTS

Detailed Methods 2

R Code for MSC meta-analysis 9

References 31

# Detailed Methods

We use a novel methodology, i.e. multiple score comparison (MSC) meta-analysis, adapted from multiple treatment effectiveness comparison network meta-analysis [1,2]. In the following sections, we introduce an explanation for each of the steps of MSC meta-analysis. More details are available on our recent statistical paper on MSC meta-analysis [3].

**MSC meta-analysis: Stage I**

The building blocks of the MSC are groups defined as the sets of cohorts for which the same prognostic scores were available. In Stage I of MSC meta-analysis, estimates are pooled within each group of cohorts, with inverse variance weighting, which accounts for the variability and number of events in each cohort.

The objective of the first stage meta‐analysis is to obtain the pooled performance difference estimate **Δ_g_**  and its variance **V_g_**≡**W^−1^_g_**  for each score group g. If there are M_g_ scores evaluated in group g, then **Δ_g_** is a (M_g_ -1) vector denoting the pooled mean performance differences for the group estimated using inverse-variance weighted least squares, and **W^−1^_g_** is a (M_g_ -1) × (M_g_ -1) matrix giving the variance of **Δ_g_**. Thus, at the end of the first stage, we have a collection of pooled data {(**Δ_g_**, **W^−1^_g_**), g=1,…, G}, that includes the direct evidence about all available comparisons.

Several methods to estimate the pooled effect in ordinary meta-analysis for two-arm trials (in our case, two-score studies) exist [4]. Less common are multi-arm trials approaches. For instance, concerning a three-score group (*M_g,j_* = 3), we can refer to the estimate vector $\boldsymbol{\Delta}_{j,g}$ regarding the individual *j-th* cohort belonging to *S_g_*, as

|  | $\boldsymbol{\Delta}_{\boldsymbol{jg}}={(\Delta_{jXY}, \Delta_{jXZ})}^{T}$ | (1) |
| --- | --- | --- |
|  |  |  |

Where Δ*_jXZ_ ,* represents the performance difference related to the score *X* and *Y* evaluated in the cohort *j* and such that the terms $\Delta_{jXY}$ and $\Delta_{jXZ}$ are evaluated as follows:

|  | $\Delta_{jYZ}={\left( -1,+1 \right)\boldsymbol{\Delta}}_{\boldsymbol{jg}}=\Delta_{jXZ}-\Delta_{jXY}$ | *(2)* |
| --- | --- | --- |

The expression for the covariance matrix is:

|  | $\boldsymbol{\Delta}_{jg}=\left( \begin{aligned} \Delta_{jXY} \\ \Delta_{jXZ} \end{aligned} \right)=\left( \begin{aligned} \Delta_{jY}- \Delta_{jX} \\ \Delta_{jZ}-\Delta_{jX} \end{aligned} \right)$*.* | *(3)* |
| --- | --- | --- |
|  |  |  |

|  | ${\boldsymbol{W}_{\boldsymbol{jg}}}^{\boldsymbol{-}\boldsymbol{1}}=var\left( \Delta_{\boldsymbol{jg}} \right)=\left( {{var(\Delta}_{jXY}) \atop{cov(\Delta}_{jXY,XZ})} {{cov(\Delta}_{jXY,XZ}) \atop{var(\Delta}_{jXZ})} \right)$ | (4) |
| --- | --- | --- |

All the terms of the covariance matrix ${\boldsymbol{W}_{\boldsymbol{jg}}}^{\boldsymbol{-1}}$ can be obtained by bootstrapping.

Given the expected heterogeneity, we used a random effect model. For instance, for the group *S_1_* we obtain:

|  | $\boldsymbol{\Delta}_{g}=\left( \Delta_{jXY}, \Delta_{jXZ} \right)^{T}=\frac{\sum_{j} \mathbf{W}_{\mathrm{jg}}\Delta_{\mathrm{jg}}}{\sum_{j} \mathbf{W}_{\mathrm{jg}}}$, | (5) |
| --- | --- | --- |
|  |  |  |

Which has the covariance matrix

|  | ${W_{jg}}^{-1}=\left( \sum_{j} W_{jg} \right)^{-1}$. | (6) |
| --- | --- | --- |
|  |  |  |

**MSC meta-analysis: Stage II**

In Stage II, the Stage I averages are combined across groups of cohorts to give overall performance estimates for the entire network. In particular, based on the direct estimates and their variances from the first stage, we determined the optimal estimate of the pooled effect parameters that obeys the fundamental consistency equations. In this stage we merged the group estimates, looking for the weighted least squares solution to the regression problem equation. We followed the approach explained and implemented in [1] and [3].

**Assessment of heterogeneity**

In the meta-analyses of direct comparisons we used a random-effects model, assuming that the true performance difference can vary according to the source population from which the study population is obtained. The heterogeneity in a direct meta-analysis indicates the variability of the true performance difference. However, the heterogeneity incorporates both true heterogeneity and also random error[4]

A similar definition can be used for the assessment of heterogeneity of network meta-analysis, adapting a definition used for multi-arm trials to multiple score comparison [1]. Since we have singleton groups in our MSC data, it is recommended in our case to use pooled estimate of the *τ^2^* (*τ^2^_pooled_*), [1] defined as follows:

$${\tau^{2}}_{pooled}=\frac{\sum_{g} Q_{g}-\sum_{g} df_{g}}{\sum_{g} C_{g}}$$

i.e. a multivariate version of the pooled estimate for the heterogeneity variance.

An alternative (not recommended in our case) is to consider not a pooled version of *τ^2^*, but a *τ^2^* for each group (*τ^2^_g_*), defined as follows:

$${\taû}_{g}^{2}=max\{0, \frac{Q_{g}-{df}_{g}}{C_{g}}\}$$

Where

$$Q_{g}=\sum_{j} {(\boldsymbol{d̂}}_{jg}-\boldsymbol{d̂}_{g})W_{g}{(\boldsymbol{d̂}}_{jg}-\boldsymbol{d̂}_{g})$$

$$df=(T_{g}-1)(N_{g}-1)$$

$$C_{g}=tr\left\{ \sum_{j} \boldsymbol{W}_{jg}-\sum_{j} \boldsymbol{W}_{jg}^{2}\left( \sum_{j} \boldsymbol{W}_{jg} \right)^{-1} \right\}$$

In the previous formulas the subscript *j* represents a generic cohort and the subscript *g* represents a generic group. Then, ***W****_jg_* is the inverse of the covariance matrix related to the performance of the scores in the cohort *j* in the group *g*. *T_g_* is the number of scores that can be evaluated in the group *g*, *N_g_* is the number of cohorts in the group *g*. Finally, **d̂_j_** is the estimate of the performance vector in the group *g* and **d̂_jg_** is the estimate of the performance vector in the cohort *j* of the group *g*.

**Assessment of transitivity**

We assessed “transitivity”, i.e., the comparability of the cohorts across whom the predictive performance of a score may vary because of a “spectrum effect”[5] or “case mix” [6–8], through ANOVA test.

For a detailed introduction to the concept of transitivity (or similarity) we recommend the suited literature [9–12]. In brief, a requirement for the application of a network meta-analytic technique is “transitivity”, i.e., in the case of treatment effect comparison, “similarity” in the distributions of “effect modifiers”[13] and in study-level characteristics (such as inclusion and exclusion criteria, details on subject recruitment, or study design choices).[8] In MSC meta-analysis we do not refer to similarity in the distributions of “effect modifiers” but to “spectrum effect”[5,14] or “case mix”,[6–8] defined as the distribution of predictor values, other relevant participant or setting characteristics (such as therapy in stable conditions) and the outcome incidence.[6] Indeed, it is well established in the prediction model literature that as the case-mix heterogeneity increases, individuals have a larger variety of patient characteristics, and the model tends to discriminate better.[15,16] In practice, we used the definition number 4 of transitivity from [12].

Thus, we evaluated by meta-regression analysis[17] the distribution of the a priori defined variables that could generate case mix variation (like median and variability of age,[6] range and variance of obstruction severity (i.e., FEV1% pred.), exercise capacity, size, mortality rate). Then, we used analysis of variance (ANOVA) to see whether the distribution of the identified variables was imbalanced in the groups and could consequently generate imbalance in the performance group by group.

We tested if case-mix heterogeneity of the following variables can affect the discriminative power of the models, namely the AUC):

Range of obstruction severity (i.e., FEV1% pred.), Variance of obstruction severity (i.e., FEV1% pred.) , mortality rate, range of exercise capacity range, median age, variability of age[6] and number of deaths.

**Assessment of inconsistency**

The above described transitivity is a requirement for consistency,[11,12] , i.e. that direct and indirect evidence do not disagree beyond what can be explained by chance. In our analysis inconsistency was defined as the disagreement of between-groups estimates (which represent the direct evidence in our methodology) and mixed score comparison meta-analysis estimates (which represent the overall evidence in our methodology). We used a test of the Q likelihood‐ratio test statistic to evaluate the overall consistency and analysis of residuals and leverages to evaluate the local consistency.[1] As already seen in a previous section of this supplementary material, we used as estimate of the heterogeneity in the MSC meta-analysis pooled heterogeneity variance among groups (*τ^2^_pooled_)*. We present the results using several numerical and graphical methods.[18].

**Global consistency**

Following the approach in literature [3], we evaluated the global consistency by likelihood-ratio test statistic, that in our case, we can express as:

|  | $Q = 2 log (\hat{L}_{H_{1}}- \hat{L}_{H_{0}})$ | (7) |
| --- | --- | --- |
|  |  |  |

With ***H_0_: d_0_ = X d_b_*** (where X represents the consistency equation and is shown below) and ***H_1_*** the observed Stage I results.

|  | $Q = \boldsymbol{y}^{\boldsymbol{T}}\boldsymbol{(W-WX}{\boldsymbol{(}\boldsymbol{X}^{\boldsymbol{T}}\boldsymbol{WX)}}^{\boldsymbol{-1}}\boldsymbol{X}^{\boldsymbol{T}}\boldsymbol{W)y}$ | (8) |
| --- | --- | --- |
|  |  |  |

Where y represents the Stage I estimates, W represents the weight matrix (inverse of the co-variance matrix), X is representing the correspondence between groups and scores or, in other words, the consistency equation:

We define *K* as the number of score assessed, *df* as the degrees of freedom (with *df=N-K+1*, where *N* is the sum of all the cores used in the groups (with this settings, N=28), *Χ^2^_df_* as the *Χ^2^* statistic with *df* degrees of freedom, 1-α as the chosen level of statistical significance. Under the null hypothesis H_0_ of global consistency, *H_0_* is of the same order of magnitude of $\chi_{N-K+1}^{2}(1-\alpha)$, i.e. the χ^2^ distribution with N-K+1 degrees of freedom and at the *1-α* level of statistical significance. In formulas:

|  | $Q\sim\chi_{N-K+1}^{2}(1-\alpha)$ | (9) |
| --- | --- | --- |
|  |  |  |

**Local consistency**

To check for local consistency we performed an analysis of residuals. In particular, we looked at the distributions of residuals and studentised residuals and Q-Q plots in fitting the consistency model in Stage II, when random-effects model is applied to Stage I.

**P-Score**

We assess the “p-score” ranking (comparable to the SUCRA score) to show the likelihood of score to be better than any other score summarizing relative performances and confidence intervals

**CHANGING THE THRESHOLD FOR MULTIPLE IMPUTATION (SENSITIVITY ANALYSIS)**

We explain here the strategy we used to handle missing data:

If a variable was missing for >30% of observations we discarded the specific variable for that particular specific cohort. If the percentage of missing data for a specific variable within a single cohort was ≤30%, we imputed the missing data (related to that variable) by multiple imputation with chained equations (the analysis of the patterns of missingness allow us to consider the missing data missing completely at random apart from the dependence on the cohort).[19–21]).

This strategy is based on the following reasoning: if the percentage of missing data for a specific variable was greater than 30%, we deemed the missing data mechanism difficult to model, meaning that an imputation technique would not have been possible to defend clinically; thus, we completely discarded the specific variable for the specific cohort. Instead, if the percentage of missing data for a specific variable within a single cohort was less than 30%, we imputed the missing data (related to that variable) by multiple imputation with chained equations[19–23].

It could be argued that the choice of 30% as threshold to decide if to discard the variable in a particular cohort or to impute it, is partly arbitrary. In part for sure it is, but it is based on same general consideration concerning missing data and on our particular analysis. First, even if, theoretically, multiple imputation techniques solve any missing data problem, as long as we correctly model the missing data mechanism, and we do not have a Missing Not At Random (MNAR) situation;[19] some techniques are suggested to impute even a variable completely missing [24]. In general, in the prediction modeling field effect of predictors with more than 50% missings in a specific data set will generally be distrusted. A more conservative threshold (say 20%) may be more acceptable in the medical field. However, our “patterns of missing data” analysis (please see the previous section on missing data) allows us to not reject the approximation that the missing data mechanism as Missing Completely at Random in each cohort (i.e. if we exclude the variable cohort). In conclusion, we used a reasonable threshold of 30%.

**EXCLUSION OF THE 2 COHORTS USED IN THE LARGE-SCALE UPDATE OF THE ADO INDEX FROM THE DATABASE (SENSITIVITY ANALYSIS)**

We included in the analyses for the main paper also the cohorts PAC-COPD and Copenhagen, even if they were used in the large-scale update of the ADO index[25]. We think it is reasonable this approach, since they are just part of the final database.

**CHECK OF THE ROBUSTNESS OF THE MSC META-ANALYSIS**

In order to check the robustness of our MSC meta-analysis, we elaborated an analysis that could include, not only the scores used in the main paper, but also the neglected original ADO[26] (and not only the updated version[25]), and also the original B-AE-D score[27] (and not only the optimized version[27]). Furthermore, we made up a score for the variables constituting the ADO score, in order to check if made-up rules could generate a good discriminative performance, and called it “ADO fake” (whose made-up scoring rules you find below).

ADO fake scoring rules:

ado.fev.fake <- cut(floor(dat$fev1pp), c(-1, 35, 50, 64, 80, 200), labels = 0:4)

ado.mmrc.fake <- cut(floor(dat$mmrc), c(-1, 0, 2, 3, 4), labels = 0:3)

ado.age.fake <- cut(dat$age_y, c(-1, 49, 59, 69, 79, 150), labels = c(0,1,13,7,9))

# R Code for MSC meta-analysis

Given the database in the following format, the main code can directly be executed:

**Database with scores**

> head(dat3)

id source1 mort GOLD new.GOLD ADO.upd BODE BODE.upd eBODE BODEx DOSE SAFE BAED.opt

6687 1 ADO_Barmelweid 0 3 NA 10 4 6 NA NA NA NA NA

6688 2 ADO_Barmelweid 1 3 NA 10 3 5 NA NA NA NA NA

6689 3 ADO_Barmelweid 0 3 NA 8 2 1 NA NA NA NA NA

6690 5 ADO_Barmelweid 0 3 NA 9 3 2 NA NA NA NA NA

6691 13 ADO_Barmelweid 1 4 NA 10 6 10 NA NA NA NA NA

6692 14 ADO_Barmelweid 1 2 NA 7 1 1 NA NA NA NA NA

**Main code**

library(pROC)

library(ggplot2)

library(boot)

library(matrixcalc) # For the function "matrix.trace()"

library(magic) # For the function "adiag()"

library(MASS) # For the function "ginv()"

setwd("~/../Desktop/NMA/")

source("MSC_functions.R")

# MSCnma() takes a dataset and evaluates AUC for each "model" with respect to "outcome", for each of the "cohorts" (all variable names in the dataset). The first model in "model" is used as a common comparator. You can choose whether to use tau^2 for each group separately (tau.pooled = FALSE), or pooled (tau.pooled = TRUE), and set the number of bootstrap samples used to estimate the variance-covariance matrix of the delta.AUCs.

# args(MSCnma)

# function (dat, model, outcome, cohorts, tau.pooled, R.boot = 100,

# seed.boot = 28121985)

load("dat3.RData")

my.dat <- dat3

# head(my.dat)

# id source1 mort GOLD new.GOLD ADO.upd BODE BODE.upd eBODE BODEx DOSE SAFE BAED.opt

# 6687 1 ADO_Barmelweid 0 3 NA 10 4 6 NA NA NA NA NA

# 6688 2 ADO_Barmelweid 1 3 NA 10 3 5 NA NA NA NA NA

# 6689 3 ADO_Barmelweid 0 3 NA 8 2 1 NA NA NA NA NA

# 6690 5 ADO_Barmelweid 0 3 NA 9 3 2 NA NA NA NA NA

# 6691 13 ADO_Barmelweid 1 4 NA 10 6 10 NA NA NA NA NA

# 6692 14 ADO_Barmelweid 1 2 NA 7 1 1 NA NA NA NA NA

scores <- c(

"GOLD",

"new.GOLD",

"ADO.upd",

"BODE",

"BODE.upd",

"eBODE",

"BODEx",

"DOSE",

"SAFE",

"BAED.opt"

)

res1 <- MSCnma(

my.dat,

model = scores,

outcome = "mort",

cohorts = "source1",

tau.pooled = FALSE,

R.boot = 50

)

res2 <- MSCnma(

my.dat,

model = scores,

outcome = "mort",

cohorts = "source1",

tau.pooled = TRUE,

R.boot = 50

)

# MSCnma() returns a list of "groupstats" (which cohorts, which scores in each group, tau^2, df and I^2%), tau2p (the pooled tau^2 value), tau.pooled (whether tau2p was used), a table of basic estimates (that is, with the common comparator only), "incons" (test for inconsistency), and "s1" (the stage 1 results)

names(res2)

# [1] "groupstats" "tau2p" "tau.pooled" "basic" "all" "incons" "s1"

res2$groupstats

# cohorts scores tau.square.g df.g I2

# B 3, 5, 20, 26 2 4.265187e-03 3 0.9173514

# C 17, 22 1, 2 2.770203e-04 2 0.1293907

# D 1, 2, 6, 7, 15 2, 3, 4 4.993448e-04 12 0.5803327

# E 4, 23, 25 1, 2, 3, 4 8.086383e-04 8 0.3772829

# F 10, 11, 13, 16, 19, 21 1, 2, 6, 7, 9 2.118093e-04 25 0.6597786

# G 18 1, 2, 3, 4, 8 8.137567e-18 0 10000

# H 9, 12, 14, 24 1, 2, 3, 4, 5, 6, 7, 9 3.174881e-04 24 0.6492014

res2$tau2p

# [1] 03548464

res2$tau.pooled

# [1] TRUE

res2$basic

# comparison d.hat SE lb ub Z p pfmt

# new.GOLD new.GOLD 0.01676424 0.007423599 0.002214249 0.03131422 2.258236 2.393098e-02 0.0239

# ADO.upd ADO.upd 0.09298788 0.006956216 0.079353944 0.10662181 13.367595 9.351614e-41 <1e-04

# BODE BODE 0.06321713 0.009074265 0.045431899 0.08100236 6.966640 3.246009e-12 <1e-04

# BODE.upd BODE.upd 0.07310281 0.010648182 0.052232759 0.09397287 6.865286 6.635837e-12 <1e-04

# eBODE eBODE 0.07009453 0.012772192 0.045061495 0.09512757 5.488058 4.063762e-08 <1e-04

# BODEx BODEx 0.05281333 0.008770087 0.035624277 0.07000239 6.021985 1.722910e-09 <1e-04

# DOSE DOSE 0.02686971 0.009295410 0.008651039 0.04508838 2.890643 3.844550e-03 0.0038

# SAFE SAFE 0.05170644 0.020360194 0.011801197 0.09161169 2.539585 1.109841e-02 0.0111

# BAED.opt BAED.opt 0.01252043 0.011419943 -0.009862251 0.03490310 1.096365 2.729191e-01 0.2729

res2$all

# comparison d.hat SE lb ub Z p pfmt

# GOLD + new.GOLD 0.01676 0.00742 0.00221 0.031314 2.2582 2.39e-02 0.02393

# GOLD + ADO.upd 0.09299 0.00696 0.07935 0.106622 13.3676 9.35e-41 < 1e-04

# GOLD + BODE 0.06322 0.00907 0.04543 0.081002 6.9666 3.25e-12 < 1e-04

# GOLD + BODE.upd 0.07310 0.01065 0.05223 0.093973 6.8653 6.64e-12 < 1e-04

# GOLD + eBODE 0.07009 0.01277 0.04506 0.095128 5.4881 4.06e-08 < 1e-04

# GOLD + BODEx 0.05281 0.00877 0.03562 0.070002 6.0220 1.72e-09 < 1e-04

# GOLD + DOSE 0.02687 0.00930 0.00865 0.045088 2.8906 3.84e-03 0.00384

# GOLD + SAFE 0.05171 0.02036 0.01180 0.091612 2.5396 1.11e-02 0.01110

# GOLD + BAED.opt 0.01252 0.01142 -0.00986 0.034903 1.0964 2.73e-01 0.27292

# new.GOLD + ADO.upd 0.07622 0.00931 0.05797 0.094476 8.1849 2.73e-16 < 1e-04

# new.GOLD + BODE 0.04645 0.01089 0.02510 0.067804 4.2642 2.01e-05 < 1e-04

# new.GOLD + BODE.upd 0.05634 0.01209 0.03264 0.080037 4.6594 3.17e-06 < 1e-04

# new.GOLD + eBODE 0.05333 0.01374 0.02639 0.080268 3.8803 1.04e-04 010

# new.GOLD + BODEx 0.03605 0.00998 0.01649 0.055606 3.6129 3.03e-04 030

# new.GOLD + DOSE 0.01011 0.01026 -0.01000 0.030212 0.9851 3.25e-01 0.32458

# new.GOLD + SAFE 0.03494 0.02136 -0.00693 0.076817 1.6355 1.02e-01 0.10195

# new.GOLD + BAED.opt -0.00424 0.01231 -0.02837 0.019885 -0.3447 7.30e-01 0.73030

# ADO.upd + BODE -0.02977 0.01025 -0.04985 -0.009687 -2.9053 3.67e-03 0.00367

# ADO.upd + BODE.upd -0.01989 0.01140 -0.04222 0.002455 -1.7446 8.11e-02 0.08105

# ADO.upd + eBODE -0.02289 0.01360 -0.04954 0.003757 -1.6837 9.22e-02 0.09224

# ADO.upd + BODEx -0.04017 0.01026 -0.06028 -0.020073 -3.9172 8.96e-05 < 1e-04

# ADO.upd + DOSE -0.06612 0.01084 -0.08736 -0.044881 -6.1019 1.05e-09 < 1e-04

# ADO.upd + SAFE -0.04128 0.02120 -0.08284 0277 -1.9469 5.15e-02 0.05154

# ADO.upd + BAED.opt -0.08047 0.01223 -0.10445 -0.056488 -6.5771 4.80e-11 < 1e-04

# BODE + BODE.upd 0.00989 0.00963 -0.00898 0.028754 1.0269 3.04e-01 0.30447

# BODE + eBODE 0.00688 0.01319 -0.01898 0.032738 0.5212 6.02e-01 0.60220

# BODE + BODEx -0.01040 0.01136 -0.03266 0.011853 -0.9162 3.60e-01 0.35958

# BODE + DOSE -0.03635 0.01192 -0.05970 -0.012990 -3.0500 2.29e-03 0.00229

# BODE + SAFE -0.01151 0.02150 -0.05365 0.030629 -0.5354 5.92e-01 0.59239

# BODE + BAED.opt -0.05070 0.01351 -0.07718 -0.024216 -3.7523 1.75e-04 018

# BODE.upd + eBODE -0.00301 0.01408 -0.03061 0.024593 -0.2136 8.31e-01 0.83084

# BODE.upd + BODEx -0.02029 0.01280 -0.04538 0.004798 -1.5851 1.13e-01 0.11294

# BODE.upd + DOSE -0.04623 0.01321 -0.07213 -0.020333 -3.4986 4.68e-04 047

# BODE.upd + SAFE -0.02140 0.02195 -0.06443 0.021633 -0.9746 3.30e-01 0.32976

# BODE.upd + BAED.opt -0.06058 0.01446 -0.08892 -0.032241 -4.1896 2.79e-05 < 1e-04

# eBODE + BODEx -0.01728 0.01331 -0.04337 0.008807 -1.2983 1.94e-01 0.19418

# eBODE + DOSE -0.04322 0.01429 -0.07124 -0.015209 -3.0239 2.50e-03 0.00250

# eBODE + SAFE -0.01839 0.02357 -0.06459 0.027818 -0.7800 4.35e-01 0.43540

# eBODE + BAED.opt -0.05757 0.01531 -0.08758 -0.027567 -3.7606 1.70e-04 017

# BODEx + DOSE -0.02594 0.01036 -0.04625 -0.005636 -2.5040 1.23e-02 0.01228

# BODEx + SAFE -0.00111 0.02194 -0.04411 0.041894 -0.0505 9.60e-01 0.95976

# BODEx + BAED.opt -0.04029 0.01126 -0.06235 -0.018232 -3.5797 3.44e-04 034

# DOSE + SAFE 0.02484 0.02217 -0.01861 0.068279 1.1205 2.62e-01 0.26249

# DOSE + BAED.opt -0.01435 0.01185 -0.03758 0.008879 -1.2108 2.26e-01 0.22598

# SAFE + BAED.opt -0.03919 0.02309 -0.08444 0.006069 -1.6971 8.97e-02 0.08967

res2$incons

# alpha K DF.Likelihood.ratio.test

# 0.0500 100 19.0000

# Q.Likelihood.ratio.test qchisq p-value

# 30.1623 30.1435 0.0498

res2$s1

# score group d.hat.s1 se.s1

# ADO.upd ADO.upd 0.1297 0.0141

# new.GOLD new.GOLD-ADO.upd 0.0158 0.0304

# ADO.upd new.GOLD-ADO.upd 0.1337 0.0442

# ADO.upd ADO.upd-BODE-BODE.upd 0.0753 0.0183

# BODE ADO.upd-BODE-BODE.upd 0.0657 0.0171

# BODE.upd ADO.upd-BODE-BODE.upd 0.0904 0.0207

# new.GOLD new.GOLD-ADO.upd-BODE-BODE.upd -0.0129 0.0225

# ADO.upd new.GOLD-ADO.upd-BODE-BODE.upd 0.0270 0.0388

# BODE new.GOLD-ADO.upd-BODE-BODE.upd 0.0429 0.0300

# BODE.upd new.GOLD-ADO.upd-BODE-BODE.upd 0.0384 0.0329

# new.GOLD new.GOLD-ADO.upd-BODEx-DOSE-BAED.opt 0.0259 0.0107

# ADO.upd new.GOLD-ADO.upd-BODEx-DOSE-BAED.opt 0.0988 0.0122

# BODEx new.GOLD-ADO.upd-BODEx-DOSE-BAED.opt 0.0584 0.0108

# DOSE new.GOLD-ADO.upd-BODEx-DOSE-BAED.opt 0.0323 0.0112

# BAED.opt new.GOLD-ADO.upd-BODEx-DOSE-BAED.opt 0.0280 0.0132

# new.GOLD new.GOLD-ADO.upd-BODE-BODE.upd-SAFE -0.0112 0.0202

# ADO.upd new.GOLD-ADO.upd-BODE-BODE.upd-SAFE 0.0367 0.0209

# BODE new.GOLD-ADO.upd-BODE-BODE.upd-SAFE 0.0498 0.0207

# BODE.upd new.GOLD-ADO.upd-BODE-BODE.upd-SAFE 0.0425 0.0217

# SAFE new.GOLD-ADO.upd-BODE-BODE.upd-SAFE 0.0412 0.0208

# new.GOLD new.GOLD-ADO.upd-BODE-BODE.upd-eBODE-BODEx-DOSE-BAED.opt 0.0259 0.0181

# ADO.upd new.GOLD-ADO.upd-BODE-BODE.upd-eBODE-BODEx-DOSE-BAED.opt 0.0866 0.0215

# BODE new.GOLD-ADO.upd-BODE-BODE.upd-eBODE-BODEx-DOSE-BAED.opt 0.0554 0.0188

# BODE.upd new.GOLD-ADO.upd-BODE-BODE.upd-eBODE-BODEx-DOSE-BAED.opt 0.0580 0.0224

# eBODE new.GOLD-ADO.upd-BODE-BODE.upd-eBODE-BODEx-DOSE-BAED.opt 0.0594 0.0185

# BODEx new.GOLD-ADO.upd-BODE-BODE.upd-eBODE-BODEx-DOSE-BAED.opt 0.0414 0.0188

# DOSE new.GOLD-ADO.upd-BODE-BODE.upd-eBODE-BODEx-DOSE-BAED.opt 0.0192 0.0197

# BAED.opt new.GOLD-ADO.upd-BODE-BODE.upd-eBODE-BODEx-DOSE-BAED.opt -0.0350 0.0278

library(ggplot2)

qplot(comparison, d.hat, data = res2$basic) +

geom_segment(aes(comparison, lb, xend = comparison, yend = ub)) +

theme_bw()

qplot(comparison, d.hat, data = res2$all) +

geom_segment(aes(comparison, lb, xend = comparison, yend = ub)) +

coord_flip() + theme_bw()

**Functions**

clinic.characteristics <- function(dat, cohorts, outcome)

{

clinic <- unique(dat[, cohorts])

clinic <- factor(clinic)

nClinics <- length(clinic)

nPatients <- nCases <- NULL

for (i in 1:nClinics) {

nPatients[i] = length(which(dat[, cohorts] == clinic[i]))

nCases[i] = length(which(dat[, cohorts] == clinic[i] &

dat[, outcome] == 1))

}

return(

list(

"clinic" = clinic,

"nClinics" = nClinics,

"nPatients" = nPatients,

"nCases" = nCases

)

)

}

AUC.scores <-

function(dat,

model,

outcome,

cohorts,

clinic,

nClinics,

nModels) {

matrix_model_cohort.AUC <-

matrix(NA,

nrow = nClinics,

ncol = nModels,

byrow = TRUE)

colnames(matrix_model_cohort.AUC) <- model

rownames(matrix_model_cohort.AUC) <- clinic

require(pROC)

for (i in 1:nClinics) {

pick.this.cohort <- which(dat[, cohorts] == clinic[i])

this.dat <- dat[pick.this.cohort,]

for (j in 1:nModels) {

this.fm <- paste(outcome, "~", model[j])

this.roc <-

try(roc(as.formula(this.fm), data = this.dat, ci = FALSE), silent = TRUE)

if (class(this.roc) == "try-error") {

matrix_model_cohort.AUC[i, j] <- NA

} else {

matrix_model_cohort.AUC[i, j] <- this.roc$auc

}

}

}

return(matrix_model_cohort.AUC)

}

Delta.AUC.scores <- function(matrix_model_cohort.AUC, nModels) {

matrix_model_cohort.DELTA.AUC <- matrix_model_cohort.AUC[,-1]

refscore <- matrix_model_cohort.AUC[, 1]

for (m in 1:(nModels - 1)) {

matrix_model_cohort.DELTA.AUC[, m] <-

matrix_model_cohort.DELTA.AUC[, m] - refscore

}

return(matrix_model_cohort.DELTA.AUC)

}

Identify.groups <- function(delta.auc, nModels, nClinics, nGroups)

{

model.per.clinic <- !is.na(delta.auc)

unique.groups <- unique(model.per.clinic)

nGroups <- nrow(unique.groups)

number.indices.per.cohort <- apply(model.per.clinic, 1, sum)

sum.NA.group <- apply(unique.groups, 1, sum)

unique.groups2 <- cbind(unique.groups, sum.NA.group)

unique.groups.ordered <-

unique.groups2[order(unique.groups2[, nModels]),]

indices.per.group <-

apply(unique.groups.ordered[, 1:(nModels - 1)], 1, function(x)

which(x == 1))

number.indices.per.group <- sapply(indices.per.group, length)

names(indices.per.group) <-

names(number.indices.per.group) <- LETTERS[1:nGroups]

number.group2 <- rep(NA, nClinics)

cohorts.per.group <- rep(list(NA), nGroups)

for (i in 1:nClinics)

{

for (g in 1:nGroups)

{

if (sum(model.per.clinic[i,] == unique.groups.ordered[g, 1:nModels - 1]) == nModels - 1)

{

number.group2[i] <- g

cohorts.per.group[[g]] <- c(cohorts.per.group[[g]], i)

}

}

}

for (i in 1:nGroups)

{

cohorts.per.group[[i]] <- cohorts.per.group[[i]][-1]

}

number.cohorts.per.group <- rep(NA, nGroups)

for (g in 1:nGroups)

{

number.cohorts.per.group[g] <- length(cohorts.per.group[[g]])

}

return(

list(

"nGroups" = nGroups,

"indices.per.group" = indices.per.group,

"number.indices.per.group" = number.indices.per.group,

"cohorts.per.group" = cohorts.per.group,

"number.cohorts.per.group" = number.cohorts.per.group

)

)

}

Initialization.variables.estimates.and.names <-

function(indices.per.group,

number.indices.per.group,

cohorts.per.group,

nSubgroups,

nGroups,

clinic)

{

NAME.subgroups.short <- rep(NA, nSubgroups)

NAME.subgroups <- rep(NA, nSubgroups)

group.estimates <- rep(list(NA), nGroups)

group.covariance.matrix <- rep(list(matrix(NA)), nGroups)

group.estimates.GROUP <- rep(list(NA), nGroups)

group.covariance.matrix.GROUP <- rep(list(matrix(NA)), nGroups)

NAME.groups <- rep(NA, nGroups)

for (g in 1:nGroups)

NAME.groups[g] <-

paste(names(indices.per.group[[g]]), collapse = "-")

NAME.cohorts.per.group <- rep(NA, nGroups)

for (g in 1:nGroups)

NAME.cohorts.per.group[g] <-

paste(clinic[cohorts.per.group[[g]]], collapse = " - ")

str.groups.description <-

cbind(NAME.groups, NAME.cohorts.per.group)

INDEX.of.SUBGROUP <- rep(NA, nSubgroups)

SUM.subgroups.up.to.group.g <- rep(NA, nGroups)

for (g in 1:nGroups)

{

SUM.subgroups.up.to.group.g[g] <-

sum(number.indices.per.group[1:g])

}

GROUP.of.SUBGROUP <- rep(NA, nSubgroups)

sub.g <- 1

for (g in 1:nGroups)

{

GROUP.of.SUBGROUP[sub.g:(sub.g + number.indices.per.group[g] - 1)] <-

g

sub.g <- sub.g + number.indices.per.group[g]

}

i2 <- indices.per.group

names(i2) <- LETTERS[1:nGroups]

NAME.subgroups <- NAME.subgroups.short <- names(unlist(i2))

INDEX.of.SUBGROUP <-

substr(NAME.subgroups, start = 3, stop = max(nchar(NAME.subgroups)))

names(indices.per.group) <- NAME.groups

names(cohorts.per.group) <- NAME.cohorts.per.group

for (g in 1:nGroups)

{

group.estimates[[g]] <- rep(NA, number.indices.per.group[g])

group.covariance.matrix[[g]] <-

matrix(NA, number.indices.per.group[g], number.indices.per.group[g])

group.estimates.GROUP[[g]] <-

rep(NA, number.indices.per.group[g])

group.covariance.matrix.GROUP[[g]] <-

matrix(NA, number.indices.per.group[g], number.indices.per.group[g])

}

names(group.estimates) <-

names(group.covariance.matrix) <- NAME.groups

names(group.estimates.GROUP) <-

names(group.covariance.matrix.GROUP) <- NAME.groups

return(

list(

"NAME.groups" = NAME.groups,

"NAME.subgroups" = NAME.subgroups,

"NAME.subgroups.short" = NAME.subgroups.short,

"groups.estimates" = group.estimates,

"group.covariance.matrix" = group.covariance.matrix,

"groups.estimates.GROUP" = group.estimates.GROUP,

"group.covariance.matrix.GROUP" = group.covariance.matrix.GROUP

)

)

}

get.delta.auc.boot.group <-

function(dat_clinic,

d,

outcome,

model,

group,

indices.per.group,

cohorts.per.group,

matrix_model_cohort.AUC)

{

dat_clinic.boot <- dat_clinic[d,]

fm0 <- paste(outcome, "~", model[1])

auc.0 <-

try(roc(as.formula(fm0), data = dat_clinic.boot, ci = TRUE), silent = TRUE)

if (class(auc.0) == "try-error") {

auc.0 = NA

} else {

auc.0 = auc.0$auc

}

AUC_I.minus.AUC0 <- rep (NA, length(model))

count <- 0

for (i in indices.per.group[[group]])

{

count <- count + 1

if (is.na(matrix_model_cohort.AUC[cohorts.per.group[[group]][1], i +

1]))

next

fm1 <- paste(outcome, "~", model[i + 1])

auc.score <-

try(roc(as.formula(fm1), data = dat_clinic.boot, ci = TRUE), silent = TRUE)

if (class(auc.score) == "try-error") {

auc.score = NA

} else {

auc.score = auc.score$auc

}

AUC_I.minus.AUC0[count] <- auc.score - auc.0

}

names(AUC_I.minus.AUC0) <- model[indices.per.group[[group]] + 1]

return (AUC_I.minus.AUC0)

}

Estimates.for.group <- function(dat,

R.boot,

seed.boot,

group,

number.indices.per.group,

number.cohorts.per.group,

indices.per.group,

cohorts.per.group,

nPatients,

cohorts,

clinic,

outcome,

model,

matrix_model_cohort.AUC)

{

group.estimates <- list(rep(NA, number.indices.per.group[group]))

group.estimates <-

rep(group.estimates, number.cohorts.per.group[group])

group.estimate.deviation.from.average <- group.estimates

group.covariance.matrix <-

list(matrix(NA, number.indices.per.group[group],

number.indices.per.group[group]))

group.covariance.matrix <-

rep(group.covariance.matrix, number.cohorts.per.group[group])

group.weights.matrix <-

list(matrix(NA, number.indices.per.group[group],

number.indices.per.group[group]))

group.weights.matrix <-

rep(group.weights.matrix, number.cohorts.per.group[group])

count <- 0

for (i in cohorts.per.group[[group]])

{

count <- count + 1

d <- c(1:nPatients[i])

dat_clinic <- subset(dat, dat[, cohorts] == clinic[i])

set.seed(seed.boot)

group.boot.i <-

boot(

dat_clinic,

get.delta.auc.boot.group,

R = R.boot,

outcome = outcome,

model = model,

indices.per.group = indices.per.group,

cohorts.per.group = cohorts.per.group,

group = group,

matrix_model_cohort.AUC = matrix_model_cohort.AUC

)

estimate <- group.boot.i$t0

covariance.matrix <-

var(group.boot.i$t, na.rm = TRUE, use = "pairwise.complete.obs")

group.estimates[[count]] <- estimate[!is.na(estimate)]

group.covariance.matrix[[count]] <-

covariance.matrix[!is.na(estimate),!is.na(estimate)]

}

for (i in 1:number.cohorts.per.group[group])

{

this.cov <- group.covariance.matrix[[i]]

group.weights.matrix[[i]] <- solve(this.cov)

}

group.weights.matrix.global <- Reduce("+", group.weights.matrix)

group.covariance.matrix.global <-

solve(group.weights.matrix.global)

SUM <- 0

for (i in 1:(number.cohorts.per.group[group]))

{

addend <-

group.covariance.matrix.global %*% group.weights.matrix[[i]] %*% group.estimates[[i]]

SUM <- SUM + addend

}

d.group <- SUM

# print("d.group")

# print(d.group)

# FIXED EFFECT ESTIMATES!!!!

Q.g.cohort <- rep(NA, number.cohorts.per.group[group])

for (i in 1:(number.cohorts.per.group[group]))

{

group.estimate.deviation.from.average[[i]] <-

group.estimates[[i]] - d.group

Q.g.cohort[i] <-

t(group.estimate.deviation.from.average[[i]]) %*% group.weights.matrix[[i]] %*% group.estimate.deviation.from.average[[i]]

}

Q <- Reduce("+", Q.g.cohort)

df <-

(number.indices.per.group[group]) * (number.cohorts.per.group[group] -

1)

group.weights.matrix.sum.square.bis <-

group.weights.matrix[[1]] ^ 2 - group.weights.matrix[[1]] ^ 2

# I do this Y = X - X to initialize the variable Y with the same dimensions of the variable X

for (i in 1:(number.cohorts.per.group[group]))

{

group.weights.matrix.sum.square.bis <-

group.weights.matrix.sum.square.bis + group.weights.matrix[[i]] %*% group.weights.matrix[[i]]

}

C <- matrix.trace(

group.weights.matrix.global -

group.weights.matrix.sum.square.bis %*% group.covariance.matrix.global

)

tau.square <- (Q - df) / C

return(list(

group.estimates,

group.covariance.matrix,

tau.square,

Q,

df,

C

))

}

Estimates.RE <-

function(group,

pooled = tau.pooled,

number.indices.per.group,

number.cohorts.per.group,

tau.square.g,

tau.square.pooled,

group.covariance.matrix,

group.estimates)

{

group.estimates.RE <- list(rep(NA, number.indices.per.group[group]))

group.estimates.RE <-

rep(group.estimates.RE, number.cohorts.per.group[group])

group.estimate.deviation.from.average.RE <- group.estimates.RE

group.covariance.matrix.RE <-

list(matrix(NA, number.indices.per.group[group],

number.indices.per.group[group]))

group.covariance.matrix.RE <-

rep(group.covariance.matrix.RE, number.cohorts.per.group[group])

group.weights.matrix.RE <-

list(matrix(NA, number.indices.per.group[group],

number.indices.per.group[group]))

group.weights.matrix.RE <-

rep(group.weights.matrix.RE, number.cohorts.per.group[group])

for (i in 1:(number.cohorts.per.group[group]))

{

if (pooled) {

tau2 <- tau.square.pooled

} else {

tau2 <- tau.square.g[group]

}

group.covariance.matrix.RE[[i]] <-

tau2 * diag(number.indices.per.group[group]) + group.covariance.matrix[[group]][[i]]

group.weights.matrix.RE[[i]] <-

solve(group.covariance.matrix.RE[[i]])

}

group.weights.matrix.global.RE <-

Reduce("+", group.weights.matrix.RE)

group.covariance.matrix.global.RE <-

solve(group.weights.matrix.global.RE)

SUM.RE <- 0

for (i in 1:(number.cohorts.per.group[group]))

{

addend <-

group.covariance.matrix.global.RE %*% group.weights.matrix.RE[[i]] %*% group.estimates[[group]][[i]]

SUM.RE <- SUM.RE + addend

}

SUM.RE

d.group.RE <- SUM.RE

d.group.RE

return(list(d.group.RE, group.covariance.matrix.global.RE))

}

Stage.2.estimate.plus.CI <- function(model,

indices.per.group,

number.indices.per.group,

group.estimates,

group.estimates.GROUP,

group.covariance.matrix.GROUP,

group.covariance.matrix,

nModels,

nGroups,

NAME.subgroups.short,

NAME.groups)

{

SUM.number.indices <- sum(number.indices.per.group)

group <- 1

X <- matrix(0, SUM.number.indices, nModels - 1)

row <- 1

for (group in 1:nGroups) {

for (j in 1:number.indices.per.group[group]) {

X[row, indices.per.group[[group]][j]] <- 1

row <- row + 1

}

}

group <- 1

Y <- rep(0, SUM.number.indices)

names(Y) <- NAME.subgroups.short

V <- do.call(adiag, group.covariance.matrix.GROUP)

W <- solve(V)

sqrt(diag(round(W, 4)))

WEIGHTS <- diag(round(W, 4))

names(WEIGHTS) <- NAME.subgroups.short

rownames(W) <- NAME.subgroups.short

colnames(W) <- NAME.subgroups.short

Y <- unlist(group.estimates.GROUP)

length(Y)

names(Y) <- NAME.subgroups.short

SE.GROUP <- lapply(group.covariance.matrix.GROUP, sqrt)

SE.GROUP.trace <- lapply(SE.GROUP, diag)

Y.SE <- unlist(SE.GROUP.trace)

WEIGHT.FINAL.pre.SOLVE <- t(X) %*% W %*% X

COVARIANCE.FINAL <- solve(t(X) %*% W %*% X)

round(ginv(t(X) %*% W %*% X), 6)

colnames(COVARIANCE.FINAL) <- unlist(model[-1])

rownames(COVARIANCE.FINAL) <- unlist(model[-1])

solve(t(X) %*% W %*% X) %*% t(X) %*% W %*% Y

round(t(X) %*% W, 0)

WEIGHTS.Y <- COVARIANCE.FINAL %*% t(X) %*% W

colnames(WEIGHTS.Y) <- NAME.subgroups.short

d.basic <- COVARIANCE.FINAL %*% t(X) %*% W %*% Y

rownames(d.basic) <- unlist(model[-1])

cbind(d.basic, sqrt(diag(COVARIANCE.FINAL)))

d.basic.Z.CI <-

qnorm(

0.975,

mean = 0,

sd = 1,

lower.tail = TRUE,

log.p = FALSE

)

diag(COVARIANCE.FINAL)

d.basic.SE <- sqrt(diag(COVARIANCE.FINAL))

d.basic.CI.low <- d.basic - d.basic.Z.CI * d.basic.SE

d.basic.CI.high <- d.basic + d.basic.Z.CI * d.basic.SE

d.basic.CI.with.CI <- cbind(d.basic, d.basic.SE)

SECOND.STAGE <- cbind(d.basic, d.basic.SE)

colnames(SECOND.STAGE) <- c("ESTIMATES", "SE")

SECOND.STAGE

### Don't initialize it during

### the different INDIRECT SIMULATIONS!!!!!!!!!!!!!!!!!!!!!!!!!!!!!!!!!!!!!

SECOND.STAGE.BASIC <- SECOND.STAGE

SECOND.STAGE <- SECOND.STAGE.BASIC

#str.final.results <- paste("NMA_results_threshold_GLOBAL", NA.score.threshold, ".xlsx", sep = "")

SECOND.STAGE.BASIC

group.estimates.BASIC <- group.estimates

names(group.covariance.matrix.GROUP) <- NAME.groups

#group.covariance.matrix.BASIC <- group.covariance.matrix

return(

list(

d.basic.CI.low,

d.basic,

d.basic.CI.high,

d.basic.SE,

Y,

X,

V,

W,

COVARIANCE.FINAL

)

)

}

Create.H <- function(model)

{

nModels <- length(model)

nCOMB.models.by.2 <- choose(nModels - 1, 2)

H.rows <- nModels - 1 + nCOMB.models.by.2

H <- matrix(0, H.rows, nModels - 1)

COMBINATIONS <- combn(nModels - 1, 2)

rownames(H) <- 1:nrow(H)

for (i in 1:nModels - 1)

{

rownames(H)[i] <- paste(c(model[1], model[i + 1]), collapse = " + ")

}

for (comb in 1:nCOMB.models.by.2)

{

rownames(H)[comb + nModels - 1] <-

paste(c(model[COMBINATIONS[, comb][1] + 1],

" + ", model[COMBINATIONS[, comb][2] +

1]), collapse = "")

}

H[1:nModels - 1, ] <- diag(nModels - 1)

for (comb in 1:choose(nModels - 1, 2))

# or length(combn(N, 2)[1,])

{

ROW <- comb + nModels - 1

H[ROW, COMBINATIONS[, comb][1]] <- -1

H[ROW, COMBINATIONS[, comb][2]] <- +1

}

return(H)

}

MSCnma <- function(dat,

model,

outcome,

cohorts,

tau.pooled,

R.boot = 100,

seed.boot = 28121985) {

nModels <- length(model)

##############################################################

##############################################################

### AUCs and DELTA.AUCs of the different SCORES

##############################################################

##############################################################

return.clinic.characteristics <-

clinic.characteristics(dat, cohorts, outcome)

clinic <- return.clinic.characteristics[[1]]

nClinics <- return.clinic.characteristics[[2]]

nPatients <- return.clinic.characteristics[[3]]

nCases <- return.clinic.characteristics[[4]]

matrix_model_cohort.AUC <-

AUC.scores(

dat,

model = model,

outcome = outcome,

cohorts = cohorts,

clinic = clinic,

nClinics = nClinics,

nModels = nModels

)

matrix_model_cohort.DELTA.AUC <-

Delta.AUC.scores(matrix_model_cohort.AUC,

nModels = nModels)

return.identify.groups <-

Identify.groups(

delta.auc = matrix_model_cohort.DELTA.AUC,

nModels = nModels,

nClinics = nClinics,

nGroups = nGroups

)

nGroups <- return.identify.groups[[1]]

indices.per.group <- return.identify.groups[[2]]

number.indices.per.group <- return.identify.groups[[3]]

cohorts.per.group <- return.identify.groups[[4]]

number.cohorts.per.group <- return.identify.groups[[5]]

nSubgroups <- sum(number.indices.per.group)

if (any(number.indices.per.group == 0)) {

excluded.groups <- which(number.indices.per.group == 0)

excluded.cohorts <- unlist(cohorts.per.group[excluded.groups])

nGroups <- nGroups - sum(excluded.groups)

indices.per.group <- indices.per.group[-excluded.groups]

number.indices.per.group <-

number.indices.per.group[-excluded.groups]

warning(

"The following cohorts have been excluded from the analysis due to lack of scores: ",

paste(clinic[excluded.cohorts], collapse = ", ")

)

cohorts.per.group <- cohorts.per.group[-excluded.groups]

number.cohorts.per.group <-

number.cohorts.per.group[-excluded.groups]

}

return.init.variables.estimates.and.names <-

Initialization.variables.estimates.and.names(

indices.per.group = indices.per.group,

number.indices.per.group = number.indices.per.group,

cohorts.per.group = cohorts.per.group,

nSubgroups = nSubgroups,

nGroups = nGroups,

clinic = clinic

)

NAME.groups <- return.init.variables.estimates.and.names[[1]]

NAME.subgroups <- return.init.variables.estimates.and.names[[2]]

NAME.subgroups.short <-

return.init.variables.estimates.and.names[[3]]

group.estimates <- return.init.variables.estimates.and.names[[4]]

group.covariance.matrix <-

return.init.variables.estimates.and.names[[5]]

group.estimates.GROUP <-

return.init.variables.estimates.and.names[[6]]

group.covariance.matrix.GROUP <-

return.init.variables.estimates.and.names[[7]]

tau.square.g <- rep(NA, nGroups)

C.g <- rep(NA, nGroups)

df.g <- rep(NA, nGroups)

Q.g <- rep(NA, nGroups)

### Fixed effect estimates

list.model <- as.list(model[-1])

for (group in 1:nGroups)

{

return.Estimates.for.group <-

Estimates.for.group(

dat,

R.boot,

seed.boot,

group,

number.indices.per.group = number.indices.per.group,

number.cohorts.per.group = number.cohorts.per.group,

indices.per.group = indices.per.group,

cohorts.per.group = cohorts.per.group,

nPatients = nPatients,

cohorts = cohorts,

clinic = clinic,

outcome = outcome,

model = model,

matrix_model_cohort.AUC = matrix_model_cohort.AUC

)

group.estimates[[group]] <- return.Estimates.for.group[[1]]

group.covariance.matrix[[group]] <-

return.Estimates.for.group[[2]]

tau.square.g[group] <- return.Estimates.for.group[[3]]

Q.g[group] <- return.Estimates.for.group[[4]]

df.g[group] <- return.Estimates.for.group[[5]]

C.g[group] <- return.Estimates.for.group[[6]]

}

tau.square.pooled <- max(0, (sum(Q.g) - sum(df.g)) / sum(C.g))

for (group in 1:nGroups)

{

return.Estimates.RE.tau <- Estimates.RE(

group,

pooled = tau.pooled,

number.indices.per.group = number.indices.per.group,

number.cohorts.per.group = number.cohorts.per.group,

tau.square.g = tau.square.g,

tau.square.pooled = tau.square.pooled,

group.covariance.matrix,

group.estimates

)

group.estimates.GROUP[[group]] <- return.Estimates.RE.tau[[1]]

group.covariance.matrix.GROUP[[group]] <-

return.Estimates.RE.tau[[2]]

}

return.Stage.2.estimate.plus.CI <-

Stage.2.estimate.plus.CI(

model,

indices.per.group,

number.indices.per.group,

group.estimates,

group.estimates.GROUP,

group.covariance.matrix.GROUP,

group.covariance.matrix,

nModels,

nGroups,

NAME.subgroups.short,

NAME.groups

)

d.basic.CI.low <- return.Stage.2.estimate.plus.CI[[1]]

d.basic <- return.Stage.2.estimate.plus.CI[[2]]

d.basic.CI.high <- return.Stage.2.estimate.plus.CI[[3]]

d.basic.SE <- return.Stage.2.estimate.plus.CI[[4]]

Y <- return.Stage.2.estimate.plus.CI[[5]]

X <- return.Stage.2.estimate.plus.CI[[6]]

V <- return.Stage.2.estimate.plus.CI[[7]]

W <- return.Stage.2.estimate.plus.CI[[8]]

COVARIANCE.FINAL <- return.Stage.2.estimate.plus.CI[[9]]

WEIGHTS.Y <- COVARIANCE.FINAL %*% t(X) %*% W

N <- sum(number.indices.per.group)

H <- Create.H(model)

d.hat <- H %*% d.basic

c_ii <- diag(H %*% COVARIANCE.FINAL %*% t(H))

d.hat.SE <- sqrt(c_ii)

d.hat.Z.CI <-

qnorm(

0.975,

mean = 0,

sd = 1,

lower.tail = TRUE,

log.p = FALSE

)

d.hat.CI.low <- d.hat - d.hat.Z.CI * d.hat.SE

d.hat.CI.high <- d.hat + d.hat.Z.CI * d.hat.SE

basic <- data.frame(

comparison = rownames(d.basic),

d.hat = d.basic,

SE = d.basic.SE,

lb = d.basic.CI.low,

ub = d.basic.CI.high,

Z = d.basic / d.basic.SE

)

basic$p <- 2 * pnorm(-abs(basic$Z))

basic$pfmt <- format.pval(basic$p, eps = 01, digits = 2)

results <- data.frame(

comparison = rownames(d.hat),

d.hat = d.hat,

SE = d.hat.SE,

lb = d.hat.CI.low,

ub = d.hat.CI.high,

Z = d.hat / d.hat.SE

)

results$p <- 2 * pnorm(-abs(results$Z))

results$pfmt <- format.pval(results$p, eps = 01, digits = 2)

#print(results, digits = 3)

#############################################################

### Inconsistency

#############################################################

Q.Likelihood.ratio.test <- t(Y) %*%

(W - W %*% X %*% COVARIANCE.FINAL %*% t(X) %*% W) %*% Y

alpha <- 0.05

K <- nModels

DF.Likelihood.ratio.test <- N - K + 1

REJECTION.NULL.HYPOTHESIS <-

Q.Likelihood.ratio.test > qchisq(1 - alpha, DF.Likelihood.ratio.test)

p.value.null.hypothesis <-

1 - pchisq(Q.Likelihood.ratio.test, DF.Likelihood.ratio.test)

INCONSISTENCY.check <-

c(

alpha,

K,

DF.Likelihood.ratio.test,

Q.Likelihood.ratio.test,

qchisq(1 - alpha, DF.Likelihood.ratio.test),

p.value.null.hypothesis

)

names(INCONSISTENCY.check) <-

c(

"alpha",

"K",

"DF.Likelihood.ratio.test",

"Q.Likelihood.ratio.test",

"qchisq",

"p-value"

)

#INCONSISTENCY.check

groupstats <-

data.frame(

"cohorts" = sapply(cohorts.per.group, paste, collapse = ", "),

"scores" = sapply(indices.per.group, paste, collapse = ", "),

tau.square.g,

df.g,

"I2" = (Q.g - df.g) / Q.g

)

tmp <-

lapply(group.covariance.matrix.GROUP, function(x)

sqrt(diag(x)))

nms <- strsplit(names(group.covariance.matrix.GROUP), "-")

s1 <- data.frame(

"score" = unlist(nms),

"group" = rep(

names(group.estimates.GROUP),

sapply(group.estimates.GROUP, length)

),

"d.hat.s1" = unlist(group.estimates.GROUP),

"se.s1" = unlist(tmp)

)

rownames(s1) <- NULL

return(

list(

"groupstats" = groupstats,

"tau2p" = tau.square.pooled,

"tau.pooled" = tau.pooled,

"basic" = basic,

"all" = results,

"incons" = INCONSISTENCY.check,

"s1" = s1

)

)

}

**Network Representation**

library(ggplot2)

datsumm <- data.frame(g = c("A", "A-C", "A-C-D-E", "A-B-C", "A-B-C-D-E", "A-B-C-D-E-F-G-H",

"A-B-C-D-E-I", "A-B-C-G-H"), n = c(162, 4313, 1195, 717, 556, 826, 4484, 3667))

allg <- sort(unique(unlist(strsplit(as.character(datsumm$g), "-")))) ## draw the points on a unit circle

angles <- seq(0, 2 * pi, length.out = length(allg) + 1) angles <- angles[angles != 2*pi] x <- cos(angles) y <- sin(angles)

# reorder summary dataset (this rearranges the groups in the plot, because the first listed is the innermost drawn, and so on out to the last listed being the outermost, or however you wish) #datsumm <- datsumm[8:1, ]

loops <- NULL for(i in 1:nrow(g)){

this.g <- which(allg %in% unlist(strsplit(as.character(datsumm$g[i]), "-"))) r <- i / 2 + nrow(datsumm)/2

this.loop <- data.frame(cohorts = i, x = r * x[this.g], y = r * y[this.g], nms = allg[this.g], n = datsumm$n[i],

deaths = datsumm$d[i])

loops <- rbind(loops, this.loop)

}

loops$cohorts <- factor(loops$cohorts) loops$nms <- as.character(loops$nms)

loops$lastone <- as.numeric(!duplicated(loops$nms, fromLast = TRUE))

ggplot(data = loops, aes(x, y)) + geom_point() + geom_label(aes(label = nms), data = subset(loops, lastone == 1), vjust = "outward", hjust = "outward") + geom_polygon(aes(color = factor(cohorts), size = n), fill = NA) + coord_equal() + guides(fill = FALSE, color = FALSE, size = FALSE) + xlim(-10, 10) + ylim(-10, 10) + theme_void()

# References

1. Lu G, Welton NJ, Higgins J, White IR, Ades AE. Linear inference for mixed treatment comparison meta-analysis: A two-stage approach. Res. Synth. Methods. 2011;2:43–60.

2. Mills EJ, Ioannidis JPA, Thorlund K, Schünemann HJ, Puhan MA, Guyatt GH. How to use an article reporting a multiple treatment comparison meta-analysis. Jama. 2012;308:1246–53.

3. Haile SR, Guerra B, Soriano JB, Puhan MA. Multiple Score Comparison: A network meta-analysis approach to comparison and external validation of prognostic scores. Stat. Methods Med. Res. BMC Medical Research Methodology; 2017;17:1–12.

4. Borenstein M, Hedges LV., Higgins J, R. RH. Introduction to Meta-Analysis. Wiley; 2011.

5. Ransohoff DF, Feinstein A. Problems of spectrum and bias in evaluating the efficacy of diagnostic tests. N. Engl. J. Med. 1978;299:926–30.

6. Riley RD, Ensor J, Snell KIE, Debray TPA, Altman DG, Moons KGM, et al. External validation of clinical prediction models using big datasets from e-health records or IPD meta-analysis: opportunities and challenges. Bmj. 2016;353:11.

7. Vergouwe Y, Moons KGM, Steyerberg EW. External validity of risk models: Use of benchmark values to disentangle a case-mix effect from incorrect coefficients. Am. J. Epidemiol. 2010;172:971–80.

8. Debray TPA, Vergouwe Y, Koffijberg H, Nieboer D, Steyerberg EW, Moons KGM. A new framework to enhance the interpretation of external validation studies of clinical prediction models. J. Clin. Epidemiol. Elsevier Inc; 2015;68:279–89.

9. Cipriani A, Higgins J, Geddes JR, Salanti G. Research and Reporting Methods Annals of Internal Medicine Conceptual and Technical Challenges in Network Meta-analysis. Ann. Intern. Med. 2013;159:130–7.

10. Donegan S, Williamson P, Gamble C, Tudur-Smith C. Indirect Comparisons: A Review of Reporting and Methodological Quality. PLoS One. 2010;5:11.

11. Song F, Loke YK, Walsh T, Glenny A-M, Eastwood AJ, Altman DG. Methodological problems in the use of indirect comparisons for evaluating healthcare interventions: survey of published systematic reviews. Bmj. 2009;338:1–7.

12. Salanti G. Indirect and mixed-treatment comparison, network, or multiple-treatments meta-analysis: many names, many benefits, many concerns for the next generation evidence synthesis tool. Res. Synth. Methods. 2012;3:80–97.

13. Dias S, Welton NJ, Sutton AJ, Caldwell DM, Lu G, Ades a E. Evidence synthesis for decision making 4: inconsistency in networks of evidence based on randomized controlled trials. Med. Decis. Making. 2013;33:641–56.

14. Mulherin SA, Miller WC. Academia and Clinic Spectrum Bias or Spectrum Effect ? Subgroup Variation in Diagnostic. Ann. Intern. Med. 2002;137:598–602.

15. Cox DR. Two Further Applications of a Model for Binary Regression. Biometrika. 1958;45:562–5.

16. Hanley JA, McNeil BJ. The Meaning and Use of the Area under a Receiver Operating ( ROC ) Curvel Characteristic. Radiology. 1982;143:29–36.

17. Thompson SG, Higgins J. How should meta-regression analyses be undertaken and interpreted? Stat. Med. 2002;21:1559–73.

18. Salanti G, Ades AE, Ioannidis JPA. Graphical methods and numerical summaries for presenting results from multiple-treatment meta-analysis: An overview and tutorial. J. Clin. Epidemiol. Elsevier Inc; 2011;64:163–71.

19. Steyerberg EW. Clinical Prediction Models. Gail M, Krickeberg K, Sarnet J, Tsiatis A, Wong W, editors. Springer - Statistics for Biology and Health - ISBN: 978-1-4419-2648-7; 2010.

20. Rubin DB. Multiple Imputation for Nonresponse in Surveys. Harvard Univ. Wiley Serie in Probability and Mathematical Statistics; 1987.

21. Harrell FE. Regression Modeling Strategies. Springer; 2015.

22. Van Buuren S, Groothuis-Oudshoorn K. Multivariate Imputation by Chained Equations. J. Stat. Softw. 2011;45:1–67.

23. Jolani S, Debray TPA, Koffijberg H, van Buuren S, Moons KGM. Imputation of systematically missing predictors in an individual participant data meta-analysis: A generalized approach using MICE. Stat. Med. 2015;34:1841–63.

24. Held U, Kessels AG, Garcia Aymerich J, Basagaña X, Ter Riet G, Moons KGM, et al. Methods for Handling Missing Variables in Risk Prediction Models. Am. J. Epidemiol. 2016;184:545–51.

25. Puhan MA, Hansel NN, Sobradillo P, Enright P, Lange P, Hickson D, et al. Large-scale international validation of the ADO index in subjects with COPD: an individual subject data analysis of 10 cohorts. BMJ Open. 2012;2:1–10.

26. Puhan MA, Garcia-Aymerich J, Frey M, ter Riet G, Antó JM, Agusti A, et al. Expansion of the prognostic assessment of patients with chronic obstructive pulmonary disease: the updated BODE index and the ADO index. Lancet. Elsevier Ltd; 2009;374:704–11.

27. Boeck L, Soriano JB, Brusse-Keizer M, Blasi F, Kostikas K, Boersma W, et al. Prognostic assessment in COPD without lung function: The B-AE-D indices. Eur. Respir. J. 2016;47:1635–44.
